# Effect of traditional Chinese medicine non-pharmacological interventions on cognitive function in patients with mild cognitive impairment: a systematic review and network meta-analysis

**DOI:** 10.3389/fmed.2026.1799759

**Published:** 2026-04-13

**Authors:** Zhihao Dong, Peizhe Li, Jing Xu, Sixian Wang, Zhijie Li, Ning Yang, Zepeng Zhang, Xingquan Wu

**Affiliations:** 1 School of Acupuncture and Tuina, Changchun University of Chinese Medicine, Changchun, China; 2 Research Center of Traditional Chinese Medicine, Affiliated Hospital of Changchun University of Chinese Medicine, Changchun, China; 3 Department of Tuina, Affiliated Hospital of Changchun University of Chinese Medicine, Changchun, China

**Keywords:** cognitive function, mild cognitive impairment, network meta-analysis, systematic review, traditional Chinese medicine non-pharmacological interventions

## Abstract

**Background:**

With the intensification of population aging, the incidence of mild cognitive impairment (MCI) continues to rise, and the risk of developing dementia is high, making it a global public health challenge. Traditional Chinese medicine non-pharmaceutical interventions (TCM-NPIs) is an important means of treating MCI. However, in clinical practice, there are various therapies, and there is no clear optimal plan yet. This study aims to evaluate the intervention effects of different TCM-NPIs on the cognitive function of MCI patients through network meta-analysis (NMA) and rank their effectiveness.

**Methods:**

The system retrieved randomized controlled trials (RCTs) related to TCM-NPIs in the treatment of MCI from 7 databases, with the search time frame from database inception to December 26, 2025. Bayesian NMA was performed on the included articles using R 4.4.3 software.

**Results:**

A total of 76 RCTs were included, involving 4,895 MCI patients and 9 types of TCM-NPIs. The NMA results showed that in terms of improving the Mini-Mental State Examination (MMSE) score effect, physical and mental exercise (MBE), massage (TN), and acupoint catgut embedding (ACE) ranked the top three. Among them, MBE (MD = 2.18, 95% CrI: 0.86 to 3.55) had the best effect, with a SUCRA of 82.56%. Regarding improvements in Montreal Cognitive Assessment (MoCA) scores, TEAS, TN, and ACE ranked in the top three, with TEAS (MD = 3.31, 95% CrI: 1.87 to 4.75) showing the best effect and a SUCRA of 88.16%. Subgroup analysis and meta-regression indicated that intervention duration and the MCI population did not have a significant impact on cognitive outcomes. In the analysis of internal difference characteristics of intervention measures, the method of moxibustion had a moderating effect on MMSE scores (*p* < 0.05), with moxibustion devices showing better efficacy (MD = 1.42, 95% CI: 0.96 to 1.87). TCM-NPIs demonstrated high safety, with no serious adverse events observed.

**Conclusion:**

Existing evidence suggests that different types of TCM-NPIs have varying therapeutic effects in improving cognitive function. Among them, MBE shows the most significant in the improvement of MMSE scores, and TEAS has the best effect in the improvement of MoCA scores.

**Systematic review registration:**

https://www.crd.york.ac.uk/PROSPERO/view/CRD420251248307, PROSPERO registration number: CRD420251248307.

## 1 Introduction

Mild cognitive impairment (MCI) is a transitional cognitive state between normal cognitive decline and early dementia. Its main features include impairment in one or more aspects of cognitive function such as memory, perception, orientation, executive function, logical reasoning, and language function, while daily living activities are relatively normal with almost no functional impairment (1). With the increasingly severe trend of population aging, the incidence of MCI is constantly rising and has become a global public health issue. The current global prevalence of MCI is approximately 19.7% (2), a proportion that increases with age and is negatively correlated with educational attainment (3). In addition, patients with MCI have a pathological change pattern similar to that of the dementia stage and are typically at an increased risk of progressing to dementia, with an annual conversion rate of 10 to 15% (4, 5), which is considerably greater than the 1–2% seen in the normal cognitive decline population (6). At present, the number of dementia patients worldwide has exceeded 55 million. Due to population aging, it is estimated that the number of patients will increase to 139 million by 2050 (7, 8). Cognitive impairment, especially neurodegenerative cognitive impairment, usually has an insidious onset and progresses slowly. Once it progresses to the dementia stage, treatment becomes difficult. Therefore, MCI is not only the precursor period of dementia, but also provides an optimal “window period” for its prevention and treatment, which can effectively reduce the conversion rate of dementia and maintain the social function of patients to the greatest extent.

Cognitive dysfunction is closely related to changes in brain structure (9), abnormal local cerebral blood flow (10), neuronal damage (11), neuroinflammation (12), and abnormal protein deposition (such as amyloid proteins and tau proteins) (13). However, no effective drug has been identified to significantly reverse the cognitive decline process of MCI. Therefore, the potential of non-pharmacological interventions (NPIs) has attracted increasing attention. Common NPIs currently available include cognitive training, dietary intervention, exercise intervention, Traditional Chinese Medicine non-pharmacological interventions (TCM-NPIs), and psychosocial therapy, etc. (6, 14, 15). Among them, TCM-NPIs have received increasing attention in improving cognitive function due to their overall regulation and multi-target effects. Manual acupuncture (MA), electroacupuncture (EA), moxibustion (MOX), tuina (TN), mind–body exercise (MBE), transcutaneous electrical acupoint stimulation (TEAS), acupoint catgut embedding (ACE), warm-needling acupuncture (WA), and auricular therapy (AT) are key components of TCM-NPIs, all of which have been widely applied in clinical practice. Several meta-analyses (16–20) have confirmed that these TCM-NPIs significantly improve cognitive function.

However, most existing studies or meta-analyses have only compared the effects between a single therapy and the control group (CON), and there is a lack of systematic comparison and summary of the relative effects and advantages of different TCM-NPIs in improving cognitive function. Therefore, this study employed a network meta-analysis (NMA) to integrate the data from randomized controlled trials (RCTS), systematically evaluating the relative efficacy of TCM-NPIs on the cognitive function of patients with MCI, in order to provide a basis for formulating more personalized and precise intervention strategies.

## 2 Methods

Our study protocol was registered with the International Prospective Register of Systematic Reviews (PROSPERO); registration number: CRD420251248307. The design and implementation of this study followed the Preferred Reporting Items for Systematic Reviews and Meta-Analyses (PRISMA) guidelines (21).

### 2.1 Search strategy

This study systematically searched the following databases: PubMed, Embase, Web of Science, Cochrane Library, CNKI, Wanfang Data, and VIP. The search time range is from the establishment of the database to December 26, 2025. Search strategy is provided in Supplementary Table 1.

### 2.2 Inclusion criteria

(1) Adults (≥18 years) diagnosed with MCI, with no restrictions on gender or ethnicity.
(2) The interventions included TCM-NPIs, such as MA, EA, MOX, TN, MBE, TEAS, ACE, WA, AT, etc.
(3) The CON may receive sham acupuncture, conventional drug treatment, maintenance of the original lifestyle, health education, or a different TCM-NPI from the treatment group.
(4) Studies included must report at least one outcome measure assessing overall cognitive function, such as Mini-Mental State Examination (MMSE), Montreal Cognitive Assessment (MoCA), etc.
(5) Study design limited to RCTs.

### 2.3 Exclusion criteria

(1) Cognitive impairment caused by other diseases (such as stroke, Parkinson’s disease, diabetes).
(2) Studies whose full text is unavailable or essential data are missing.
(3) Duplicate publications or reports with overlapping data.
(4) There was no difference in the application of TCM-NPIs between groups.

### 2.4 Study selection and data extraction

Literature screening and data extraction were independently completed by two researchers (JX and SW) and checked against each other. If there were any differences, a third researcher (NY) would assist in resolving them. During the literature screening process, duplicate studies were first removed through duplicate detection. Then, the titles and abstracts of the remaining studies were reviewed. After excluding those clearly irrelevant, the full texts were further assessed to determine whether they should be included. The extracted information includes: first author, publication year, country, average age, sample size of each group, intervention measures, treatment period, outcome indicators and result measurement data, etc.

### 2.5 Risk of bias and quality of evidence assessment

Risk of bias and evidence quality were independently assessed and cross-checked by two researchers (JX and SW) using the Cochrane Risk of Bias tool version 2.0 (RoB 2.0) and CINeMA (Confidence in Network Meta-Analysis) (22). The specific assessment dimensions are detailed in Supplementary Table 2. Disagreements were resolved by consulting a third researcher (NY).

### 2.6 Statistical analysis

This study conducted a Bayesian network meta-analysis using the gemtc package in R software (version 4.4.3). The outcome measures for statistical analysis were all continuous variables, with mean difference (MD) used as the effect size. A 95% credible interval (95% CrI) excluding zero was considered indicative of a significant difference. Given the variations in intervention approaches across different TCM-NPIs, a random-effects model was applied to account for potential heterogeneity between studies. NMA adopts the Markov Chain Monte Carlo method for parameter estimation and is implemented based on the JAGS sampler. A total of 4 Markov chains are set up, and each chain undergoes 25,000 iterations. Among them, the first 5,000 are used as the burn-in period, and the last 20,000 are used for posterior inference. The Potential Scale Reduction Factor (PSRF) reflects convergence. A PSRF value closer to 1 indicates better convergence performance. The relationships among the various intervention measures are represented through the evidence network. Global consistency is evaluated by comparing the Deviance Information Criterion (DIC) between the consistency model and the inconsistency model. When the DIC difference is greater than 5, it indicates that there may be potential inconsistencies. The local inconsistency test adopts the node-splitting method. A *p* > 0.05 indicates good consistency. The comparison results among the various intervention measures are presented through the league table. The intervention measures were ranked by the Surface Under the Cumulative Ranking Curve (SUCRA), and the value of SUCRA (0% ≤ SUCRA ≤ 100%) was positively correlated with the intervention effect. In addition, this study took the MCI population and the intervention cycle as covariates to conduct subgroup analysis and meta-regression of NMA, and paired analysis was carried out on the intervention characteristics within each intervention measure (such as needle retention time) to evaluate their moderating effects on the intervention outcome.

## 3 Results

### 3.1 Study selection

**Figure 1:**
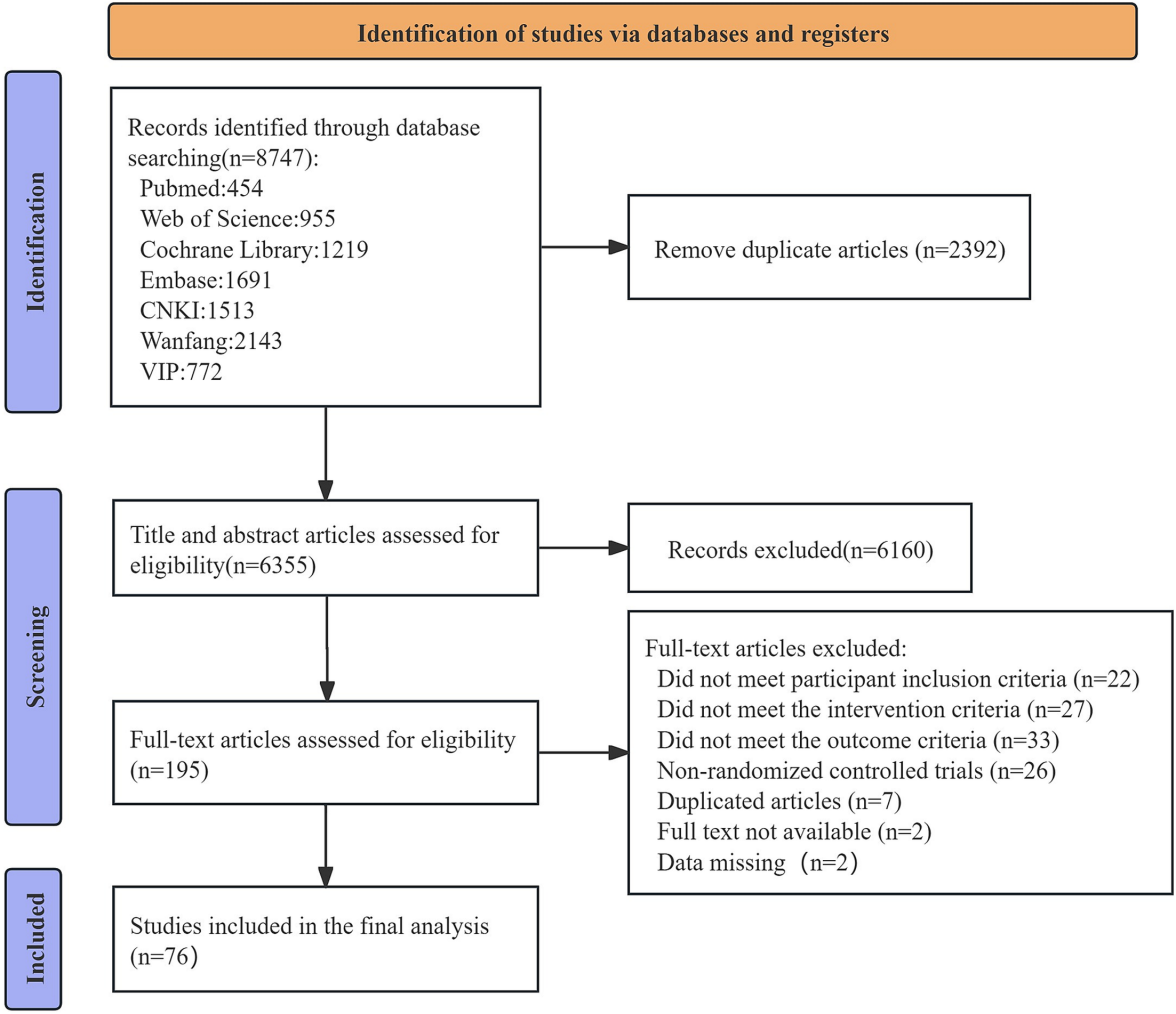
Literature selection flowchart.

A total of 8,747 relevant documents were retrieved through the search. After plagiarism checks and preliminary screening, 8,552 documents were excluded. Following full-text reading of the remaining 195 documents, 76 qualified documents were ultimately included (see Figure 1).

### 3.2 Characteristics of the included studies

A total of 76 articles (23–98) published from 2009 to 2025 were included in this study, involving 4,895 MCI patients, with 2,472 in the experimental group and 2,423 in the CON. The majority of the research subjects are elderly people over 60 years old. Except for two studies which were conducted in the United States and South Korea respectively, the rest were all carried out in China. The intervention methods include a total of 9 TCM-NPIs: MA, EA, MOX, TN, MBE, TEAS, ACE, WA, and AT. The specific definition of the intervention method can be found in Supplementary Table 3. One study (53) is a direct comparison between MA and ACE, while the rest are comparisons between TCM-NPIs and conventional controls (see Supplementary Table 4).

### 3.3 Results of risk of bias and quality of evidence assessment

Among of the 76 studies, 63.2% were evaluated as low risk of bias, 32.9% as some concerns, and 3.9% as high risk of bias (Supplementary Figures 1, 2). Since many studies have not described the random allocation method in detail, and the particularity of TCM-NPIs makes it difficult to implement blinding, this may affect the evaluation results.

Based on the evidence quality assessment results (Supplementary Table 5) from CINeMA, 36 pairwise comparisons were formed for the MMSE score, with 16.7% of comparisons showing high confidence, 47.2% moderate confidence, and 36.1% low confidence. For the MoCA score, 45 pairwise comparisons were made, with 6.7% showing low confidence and the rest being very low. The low confidence was primarily due to the incoherence domain, where all comparisons were rated as Major concerns. The network structure of MoCA lacked a closed loop, making it impossible to assess the incoherence between different pieces of evidence. According to the CINeMA evaluation system, incoherence was classified as Major concerns, which lowered the overall credibility level of the evidence.

### 3.4 NMA results

#### 3.4.1 MMSE

**Figure 2:**
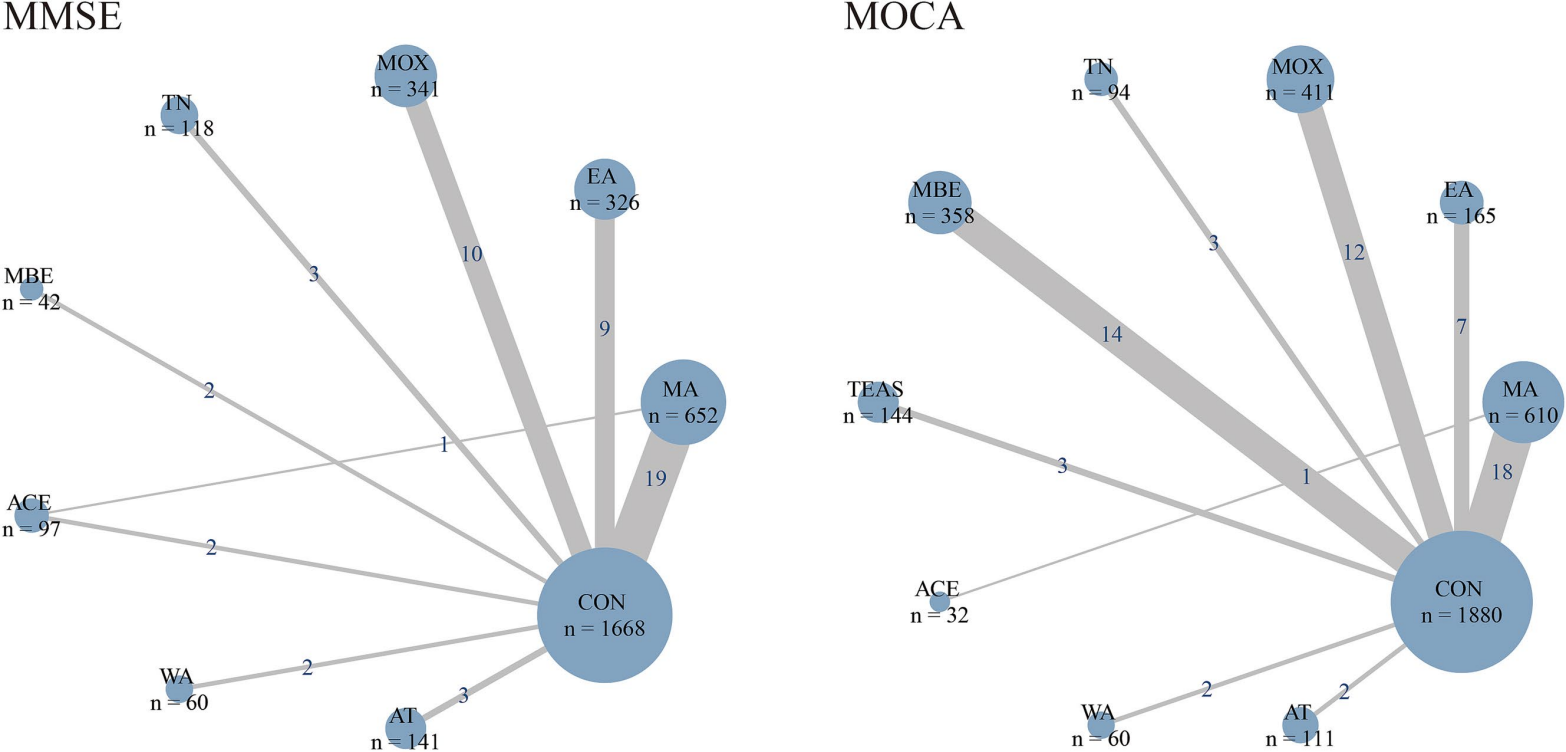
Network plot of evidence. The width of the lines represents the number of studies between each pair of interventions, and the size of the nodes reflects the cumulative sample size for each intervention. MA, manual acupuncture; EA, electroacupuncture; MOX, moxibustion; TN, tuina; MBE, mind–body exercise; TEAS, transcutaneous electrical acupoint stimulation; ACE, acupoint catgut embedding; WA, warm-needling acupuncture; AT, auricular therapy; CON, control group.

A total of 51 articles reported changes in MMSE scores before and after intervention, including 3,445 MCI patients. The model demonstrated good convergence (Supplementary Figure 3). The network evidence diagram is shown in Figure 2, which includes 8 types of TCM-NPIs: MA, EA, MOX, TN, MBE, ACE, WA, and AT. All interventions are two-arm comparisons, with a direct comparison between MA and ACE, forming a closed loop with CON. Node-splitting analysis results showed that the direct effect between ACE and MA was 0.93 (95% CrI: −0.69 to 2.58), and the indirect effect was −0.35 (95% CrI: −1.51 to 0.93). The inconsistency test showed that the *p*-values for the pairwise comparisons between the three groups were all greater than 0.05, indicating no significant inconsistency was found in this comparison (Supplementary Figure 4). In addition, the sample sizes of MA, MOX, and EA are relatively large, and there are more literatures directly comparing them with CON, making the network evidence more reliable.

Eight types of TCM-NPIs were effective in improving MMSE scores in MCI patients compared to CON. Among them, MBE (MD = 2.18, 95% CrI: 0.86 to 3.55) showed the most significant improvement, demonstrating a clear advantage. Following that, TN (MD = 1.98, 95% CrI: 1.06 to 2.9), ACE (MD = 1.86, 95% CrI: 0.9 to 2.82), MA (MD = 1.75, 95% CrI: 1.38 to 2.12), AT (MD = 1.2, 95% CrI: 0.35 to 2.07), EA (MD = 1.19, 95% CrI: 0.66 to 1.71), WA (MD = 1.14, 95% CrI: 0.09 to 2.19), MOX (MD = 1.14, 95% CrI: 0.09 to 2.19), (MD = 1.14, 95% CrI: 0.09 to 2.19 1.19, 95% CrI: 0.66 to 1.71), WA (MD = 1.14, 95% CrI: 0.09 to 2.19), and MOX (MD = 1.07, 95% CrI: 0.57 to 1.57) also demonstrated good intervention effects (Table 1; Supplementary Figure 5).

**Table 1:** League table of intervention comparisons.

Ma	EA	MOX	TN	MBE	TEAS	ACE	WA	AT	CON
MA	0.56 (−0.08, 1.21)	**0.68 (0.06, 1.31)**	−0.23 (−1.22, 0.76)	−0.43 (−1.85, 0.94)	–	−0.11 (−1.09, 0.87)	0.61 (−0.5, 1.72)	0.55 (−0.39, 1.47)	**1.75 (1.38, 2.12)**
**1.39 (0.25, 2.54)**	EA	0.12 (−0.6, 0.84)	−0.79 (−1.86, 0.27)	−0.99 (−2.46, 0.43)	–	−0.67 (−1.76, 0.41)	0.05 (−1.13, 1.23)	−0.01 (−1.02, 0.99)	**1.19 (0.66, 1.71)**
0.38 (−0.56, 1.32)	−1.02 (−2.25, 0.21)	MOX	−0.91 (−1.97, 0.15)	−1.11 (−2.58, 0.3)	–	−0.79 (−1.87, 0.29)	−0.07 (−1.23, 1.1)	−0.14 (−1.13, 0.86)	**1.07 (0.57, 1.57)**
−0.8 (−2.35, 0.74)	**−2.19 (−3.94, −0.45)**	−1.18 (−2.79, 0.43)	TN	−0.2 (−1.87, 1.41)	–	0.12 (−1.21, 1.45)	0.84 (−0.56, 2.24)	0.78 (−0.5, 2.04)	**1.98 (1.06, 2.9)**
0.1 (−0.83, 1.02)	**−1.3 (−2.51, −0.09)**	−0.28 (−1.32, 0.74)	0.89 (−0.7, 2.5)	MBE	–	0.32 (−1.3, 2)	1.04 (−0.64, 2.77)	0.98 (−0.59, 2.59)	**2.18 (0.86, 3.55)**
−1.13 (−2.67, 0.42)	**−2.52 (−4.27, −0.79)**	−1.51 (−3.12, 0.1)	−0.34 (−2.35, 1.71)	−1.22 (−2.83, 0.39)	TEAS	–	–	–	–
−1.05 (−3.42, 1.32)	−2.45 (−5.07, 0.18)	−1.43 (−3.98, 1.12)	−0.26 (−3.08, 2.56)	−1.14 (−3.69, 1.39)	0.08 (−2.75, 2.9)	ACE	0.72 (−0.7, 2.14)	0.65 (−0.64, 1.93)	**1.86 (0.9, 2.82)**
1.37 (−0.38, 3.12)	−0.03 (−1.95, 1.9)	0.99 (−0.82, 2.8)	**2.16 (0, 4.35)**	1.27 (−0.5, 3.07)	**2.5 (0.31, 4.67)**	2.42 (−0.52, 5.36)	WA	−0.06 (−1.42, 1.28)	**1.14 (0.09, 2.19)**
1.05 (−0.71, 2.84)	−0.35 (−2.29, 1.61)	0.67 (−1.15, 2.52)	1.84 (−0.34, 4.07)	0.96 (−0.85, 2.78)	2.18 (−0.02, 4.39)	2.1 (−0.86, 5.08)	−0.31 (−2.66, 2.03)	AT	**1.2 (0.35, 2.07)**
**2.18 (1.61, 2.77)**	0.79 (−0.19, 1.78)	**1.8 (1.07, 2.55)**	**2.98 (1.55, 4.43)**	**2.09 (1.38, 2.81)**	**3.31 (1.87, 4.75)**	**3.24 (0.8, 5.67)**	0.82 (−0.83, 2.47)	1.13 (−0.54, 2.8)	CON

The upper-right triangle corresponds to MMSE outcomes (row vs. column), while the lower-left triangle corresponds to MoCA outcomes (column vs. row). Statistically significant results are highlighted in bold. MA, manual acupuncture; EA, electroacupuncture; MOX, moxibustion; TN, tuina; MBE, mind–body exercise; TEAS, transcutaneous electrical acupoint stimulation; ACE, acupoint catgut embedding; WA, warm-needling acupuncture; AT, auricular therapy; CON, control group.

**Figure 3:**
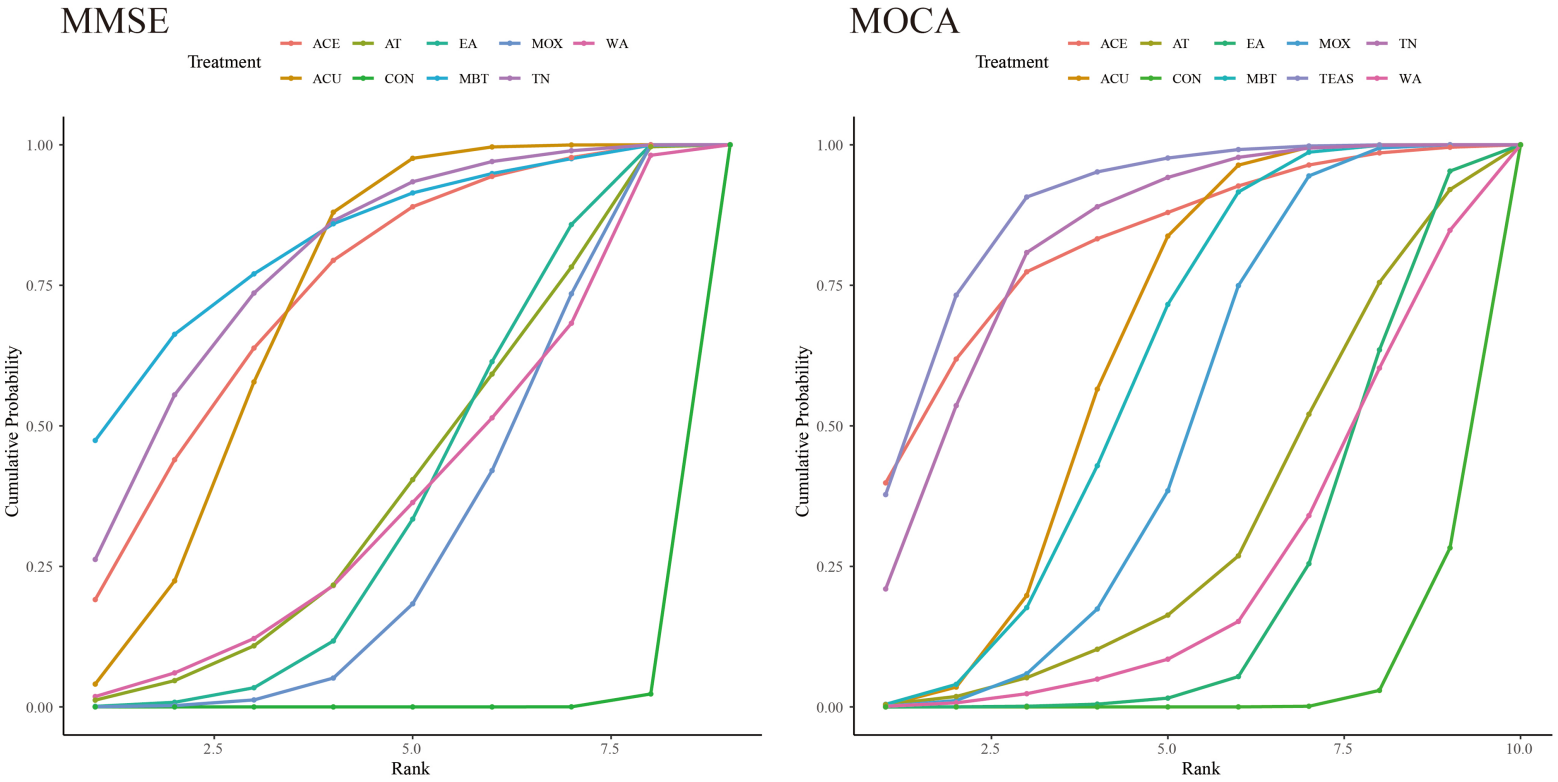
Cumulative probability plot. MA, manual acupuncture; EA, electroacupuncture; MOX, moxibustion; TN, tuina; MBE, mind–body exercise; TEAS, transcutaneous electrical acupoint stimulation; ACE, acupoint catgut embedding; WA, warm-needling acupuncture; AT, auricular therapy; CON, control group.

The SUCRA ranking results (Table 2) showed: MBE (82.56%) > TN (78.91%) > ACE (73.73%) > MA (71.18%) > EA (37.09%) > WA (36.98%) > MOX (30.07%) > AT (3.95%) > CON (0.29%). The cumulative probability plot (Figure 3) indicated that MBE had the largest area under the curve, suggesting that MBE was superior to other interventions in improving MMSE scores.

**Table 2:** SUCRA rankings of interventions for cognitive outcomes.

Rank	MMSE	SUCRA (%)	MoCA	SUCRA (%)
1	MBE	82.56	TEAS	88.16
2	TN	78.91	ACE	81.95
3	ACE	73.73	TN	81.74
4	MA	71.18	MA	62.20
5	EA	37.09	MBE	58.54
6	WA	36.98	MOX	47.98
7	MOX	30.07	AT	31.17
8	AT	3.95	WA	23.44
9	CON	0.29	EA	21.32
10	–	–	CON	3.48

MA, manual acupuncture; EA, electroacupuncture; MOX, moxibustion; TN, tuina; MBE, mind–body exercise; TEAS, transcutaneous electrical acupoint stimulation; ACE, acupoint catgut embedding; WA, warm-needling acupuncture; AT, auricular therapy; CON, control group.

#### 3.4.2 MoCA

A total of 62 articles reported changes in MoCA scores before and after intervention, including 3,865 MCI patients. The model demonstrated good convergence (Supplementary Figure 6). The network evidence diagram is shown in Figure 2, which includes 9 types of TCM-NPIs. All interventions, except ACE, were compared only with CON, and there was a direct comparison between ACE and MA. Since the network structure did not form a closed loop, no local inconsistency test was performed. The sample sizes of MA, MBE, MOX and EA are relatively large, and there are many literatures directly comparing them with CON. Therefore, the evidence for these intervention measures is more reliable.

MA, MOX, TN, MBE, TEAS, and ACE therapies all effectively improve MoCA scores compared to CON, while EA, WA, and AT show no significant improvement. TEAS (MD = 3.31, 95% CrI: 1.87 to 4.75) showed the most significant improvement, demonstrating a clear advantage. Following that, ACE (MD = 3.24, 95% CrI: 0.8 to 5.67), TN (MD = 2.98, 95% CrI: 1.55 to 4.43), MA (MD = 2.18, 95% CrI: 1.61 to 2.77), MBE (MD = 2.09, 95% CrI:1.38 to 2.81), and MOX (MD = 1.8, 95% CrI:1.07 to 2.55) also showed good intervention effects (Table 1; Supplementary Figure 7).

The SUCRA ranking results (Table 2) showed: TEAS (88.16%) > ACE (81.95%) > TN (81.74%) > MA (62.20%) > MBE (58.54%) > MOX (47.98%) > AT (31.17%) > WA (23.44%) > EA (21.32%) > CON (3.48%). The cumulative probability plot (Figure 3) showed that TEAS had the largest area under the curve, suggesting that TEAS was superior to other intervention methods in improving the MoCA score.

### 3.5 Publication bias analysis

The scatter distribution on the MMSE funnel plot shows asymmetry, and Egger’s test (*p* = 0.0242) suggests potential bias. In contrast, the distribution of the MoCA funnel plot appeared more symmetrical, and the Egger test (*p* = 0.1141) did not detect significant bias, as shown in Supplementary Figure 8.

## 4 NMA subgroup analysis and meta-regression

### 4.1 Subgroup analysis by intervention duration

Intervention duration, as a potential moderating factor, may affect the improvement of cognitive function by interventions. Therefore, in this study, the intervention duration in the included studies was categorized into short-term (≤8 weeks), medium-term (8 weeks < duration <24 weeks), and long-term (≥24 weeks). Subgroup analysis of MMSE scores showed that ACE (MD = 2.03, 95% CrI: 0.45 to 3.62), MBE (MD = 2.21, 95% CrI: 0.85 to 3.62), MA (MD = 2.44, 95% CrI: 0.86 to 3.89) demonstrated the most significant improvement in short-term, medium-term, and long-term interventions, respectively. For MoCA scores, ACE (MD = 3.49, 95% CrI: 1.05 to 5.94), TEAS (MD = 3.32, 95% CrI: 1.9 to 4.72), TN (MD = 3.86, 95% CrI: 2.11 to 5.56) showed the best results in short-term, medium-term, and long-term interventions, respectively. MBE and TEAS were most effective during medium-term interventions. See Supplementary Tables 6–10 and Supplementary Figures 9, 10 for details. Meta-regression results indicated that the intervention duration did not have a significant moderating effect on cognitive outcomes (Supplementary Figures 11, 12).

### 4.2 Subgroup analysis by MCI population type

In this study, patients in 18 studies (28, 32–35, 37–39, 43–46, 55, 58, 64, 77, 81, 86) were definitively diagnosed with amnestic MCI (aMCI), while the remaining studies merely classified participants as having MCI without specifying a specific subtype. Based on this, the present study categorizes MCI patients into two categories: aMCI and unspecified MCI (uMCI). Given the uncertainty regarding subtypes in the uMCI group and the potential for heterogeneity, this study analyzed results only for the aMCI group, while results for the uMCI group are provided for exploratory reference only. The results showed that among aMCI patients, MOX (MD = 2.18, 95% CrI: 1.29 to 3.08) and TEAS (MD = 3.04, 95% CrI: 1.21 to 4.85) were more effective in improving MMSE and MoCA scores, respectively. See Supplementary Tables 11–14 and Supplementary Figures 13, 14 for details. Meta-regression results showed that the MCI population did not have a significant impact on cognitive outcomes (Supplementary Figures 15, 16).

## 5 Analysis of internal differences in intervention measures

This study systematically analyzed the internal differential characteristics of various intervention measures, including MA retention time, EA waveform and duration, MOX application methods and duration, and types of MBE. Other intervention methods were not included in the analysis due to limitations in the available literature. Subgroup analysis and meta-regression indicated that MOX devices (MD = 1.42, 95% CI: 0.96 to 1.87) were more effective than traditional ignited MOX (MD = 0.46, 95% CI: 0.24 to 0.67) and had a significant moderating effect on MMSE scores (*p* < 0.05). Other internal differences did not reach statistical significance in cognitive function improvement (Supplementary Figures 17–36).

## 6 Adverse events

Among the 76 studies included, 36 reported the occurrence of adverse events, with 22 studies (24–26, 34, 37, 39, 42, 48, 52, 55, 57, 61, 65, 66, 77–80, 82, 85, 91, 96) explicitly stating that no adverse events occurred. The remaining 14 studies (32, 36, 38, 44, 45, 47, 49, 58, 68, 72, 73, 90, 92, 95) reported common minor adverse reactions related to the interventions, including MA, EA, MOX, MBE, TEAS, and AT. These adverse events include a small amount of subcutaneous bleeding caused by MA, EA and AT, dizziness caused by TEAS, minor skin burns caused by MOX, muscle discomfort and pain caused by MBE, etc.

## 7 Discussion

### 7.1 Summary of main findings

This study used a NMA to systematically evaluate the effects of nine categories of TCM-NPIs on the overall cognitive function of patients with MCI. The results showed that MBE (MD = 2.18, SUCRA = 82.56%) demonstrated the greatest effect in improving MMSE scores, while TEAS (MD = 3.31, SUCRA = 88.16%) performed better on the MoCA, suggesting that TCM-NPIs may have therapeutic differences in different cognitive dimensions. Subgroup analysis and meta-regression indicated that neither the duration of intervention nor the MCI population exerted a significant moderating effect on treatment outcomes. Among the internal differences in intervention measures, the application method of moxibustion influenced its efficacy, with moxibustion devices yielding better results.

### 7.2 Interpretation of results and possible mechanisms

The differences in the effects of TCM-NPIs on the MMSE and MoCA scores may be closely related to the sensitivity of the scales’ cognitive dimensions and the mechanisms of action of the interventions.

The MMSE focuses on the assessment of basic cognition, including orientation, attention, memory, language function, etc. (99). Its task design is relatively simple and prone to the ceiling effect (100). MBE, combining physical exercise, breathing regulation, and mental focus, broadly stimulates the central nervous system and enhances cognitive networks, thereby providing functional support for improvements in MMSE scores (101, 102). Neuroimaging studies have shown that MBE can modulate the default mode network and regulate interregional connectivity in the brain. For example, Tai Chi practice can significantly enhance resting-state functional connectivity between the posterior cingulate cortex and the putamen/caudate (core structures of the basal ganglia), thereby maintaining the neural networks essential for basic cognitive functions (103). The basal ganglia, as a key subcortical node, are involved in MMSE-related cognitive processes such as memory consolidation, attention regulation, and language function (104, 105). In addition, after Baduanjin training, the gray matter volume in the temporal lobe, cingulate gyrus and occipital lobe of MCI patients increased significantly. These areas are closely related to immediate memory encoding, basic language function, orientation maintenance, etc. (60, 106). In terms of molecular mechanisms, MBE can increase levels of brain-derived neurotrophic factor (BDNF) in the brain. Its complex motor components may promote BDNF expression through the muscle-brain axis, specifically the peroxisome proliferator-activated receptor gamma coactivator 1-alpha (PGC-1α)/fibronectin type III domain-containing protein 5 (FNDC5)/BDNF pathway (107). The psychological regulation component can activate neurons in brain regions to release glutamate, which, through the N-methyl-D-aspartate receptor (NMDA)/Ca^2+^/cAMP response element-binding protein (CREB) cascade, promotes BDNF gene transcription. BDNF enhances synaptic plasticity, particularly promoting the improvement of memory and learning abilities in the hippocampus and cortex (108, 109). Further basic research confirmed that specific knockout of hippocampal BDNF severely impaired spatial working memory and synaptic transmission in mice, which could be restored by exogenous BDNF administration (110).

The MoCA provides a more comprehensive and in-depth assessment of cognitive functions, such as executive function, visuospatial abilities, and abstract thinking. In identifying MCI, the MoCA has a sensitivity of 80 to 100% (111). TEAS can significantly enhances the function of the prefrontal cortex (PFC) by applying electrical pulses via electrodes placed on acupoints associated with cognitive functions (112, 113). The PFC is the core neural basis for executive function, working memory, and abstract thinking (114). In particular, the dorsolateral prefrontal cortex (dlPFC) is a central hub for higher-order cognitive processing (115) and a primary target region for non-invasive brain stimulation (116). The core sub-items detected by MoCA are closely related to the function of PFC. Meta-analysis evidence suggests that electrical stimulation of the dlPFC produces cognitive improvements with a large effect size (SMD = 0.49, *p* < 0.0001), and that stimulation of the dlPFC enhances executive function, memory processing, and other cognitive functions (117, 118). Neuroimaging studies have further confirmed that dlPFC stimulation enhances local neural electrical activity and cerebral blood perfusion, thereby improving cognitive control, attention regulation, and advanced executive functions (114, 119). From a neurophysiological perspective, TEAS significantly increases local oxygenated hemoglobin levels and cerebral blood flow in the PFC, providing essential metabolic support to active neurons (120). Concurrently, acupoint electrical stimulation significantly activates the BDNF/tyrosine kinase receptor B (TrkB) pathway in the PFC, thereby significantly enhancing synaptic plasticity and increasing information processing capacity, providing a molecular basis for improved cognitive function (121–123).

In conclusion, the differences in the effects of MBE and TEAS on MMSE and MoCA may reflect the differences in the cognitive domains evaluated by the scales and the different intervention paths. At present, there is still a lack of clear evidence regarding the specific action pathways and causal relationships of MBE and TEAS. Future research should combine neuroimaging, molecular biology, and cognitive subfields to enhance the evidence level of intervention-mechanism-cognitive effects.

### 7.3 Comparison with previous studies

Previous studies also support the main findings of this research. Multiple meta-analyses have shown that MBE interventions represented by Tai Chi and Baduanjin can significantly enhance the attention, learning and memory, language ability, etc. of MCI patients, thereby improving the MMSE score (124–126). Wu et al. (127) further pointed out that the intervention effect of MBE on cognitively impaired individuals is significantly better than that on cognitively normal individuals. Its effect is more targeted and sensitive, and the movements are gentle and the body and mind are coordinated. Low to moderate intensity training can lead to cognitive improvement (128, 129). Furthermore, existing meta-analyses have shown that TEAS can effectively improve MoCA scores in patients with cognitive impairment (130–132). Although most existing studies have focused on postoperative cognitive impairment, the results of this study, when combined with the existing evidence, indicate that TEAS also has a cognitive-enhancing effect in non-postoperative MCI patients. Subgroup analyses revealed that MBE and TEAS were most effective during the mid-intervention phase, consistent with the findings of previous meta-analyses on non-pharmacological treatments for MCI, which indicated that clinically meaningful cognitive benefits can be achieved as early as the mid-intervention phase (133).

### 7.4 Limitations

Although the results of this study have certain implications, there are still several limitations. Firstly, the number of literature on some intervention methods is limited, and the overall research quality varies. Due to the difficulty in implementing blinding in TCM-NPIs and the insufficiently described randomization methods in some studies, only 63.2% of the studies were evaluated as having a low risk of bias. This may affect the accuracy of the intervention effect evaluation and thereby weaken the robustness of the conclusion. For instance, insufficient randomization may lead to uneven baseline cognitive levels, and incomplete blinding implementation may cause expectation bias in evaluators or subjects, affecting outcome measurement. Secondly, this study used the global cognitive scale as the outcome indicator. Due to the limitations of subdomain data, no analysis was conducted on specific cognitive subdomains (such as memory, executive function, attention, and language), which might have underestimated the advantages of certain interventions in specific cognitive dimensions. Furthermore, the diagnosis of MCI subtypes in the included RCTs was not clear enough. Only some studies clearly identified them as aMCI, while the remaining studies did not distinguish specific subtypes. This might have increased the heterogeneity among studies and limited the applicability of the results in clinical practice. Future research should strictly follow all the standards for the implementation of RCT, clearly distinguish the subtypes of MCI, and conduct detailed evaluations in combination with each subfield of cognition to improve the accuracy and clinical applicability of intervention effect assessment.

## 8 Conclusion

The results of this study indicate that not all TCM-NPIs have significant therapeutic effects in improving the cognitive level of patients with MCI. Specifically, MBE has the best effect in improving MMSE scores, and TEAS has more advantages in enhancing MoCA. This suggests that TCM-NPIs may achieve the improvement of cognitive function by acting on different cognitive dimensions. This study provides evidence-based intervention strategies for MCI patients and offers a reference for the clinical selection of appropriate intervention methods. However, due to limitations such as the quality of literature, insufficient data in subfields, and unclear diagnosis of MCI subtypes, these conclusions still need to be further verified through high-quality, multi-center RCTs to optimize intervention plans and guide clinical practice.

## Data Availability

The original contributions presented in the study are included in the article/Supplementary material, further inquiries can be directed to the corresponding authors.
