# Supplementary material for: Effect of traditional Chinese medicine non-pharmacological interventions on cognitive function in patients with mild cognitive impairment: a systematic review and network meta-analysis
Source: Front Med (Lausanne). 2026 Apr 13;13:1799759. doi: 10.3389/fmed.2026.1799759 (PMC13110958; doi:10.3389/fmed.2026.1799759)
Supplement: Supplementary file 1 [file Data_Sheet_1.docx]

Supplementary Material

# Supplementary Figures and Tables

## **1.1 Supplementary Figures**


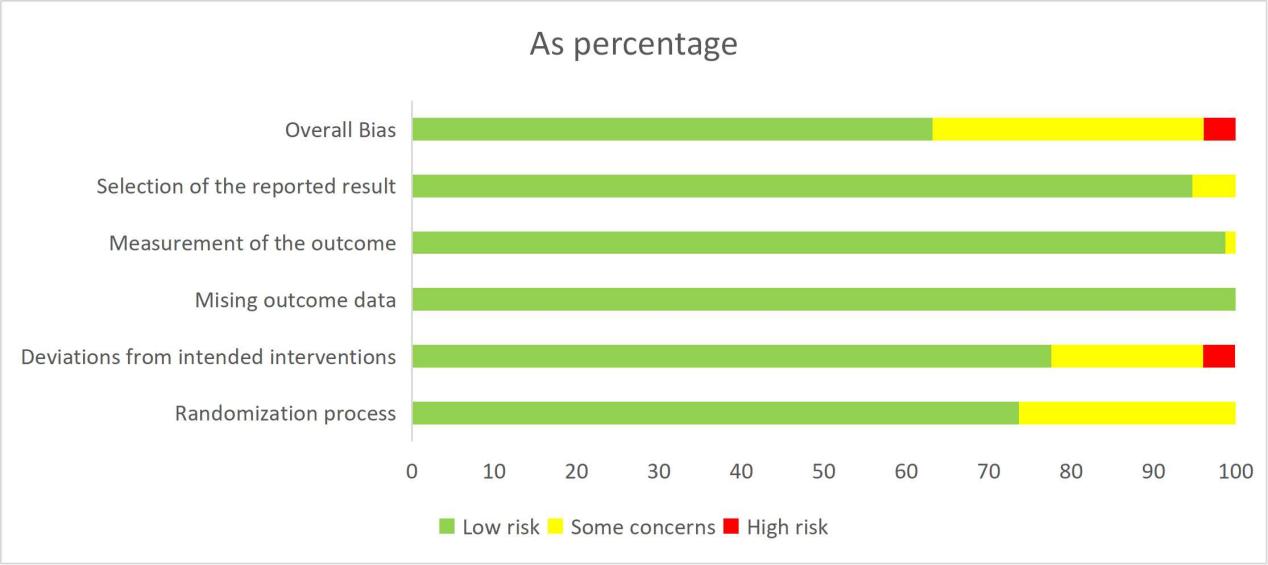


**Supplementary Figure 1** Summary of bias risk.


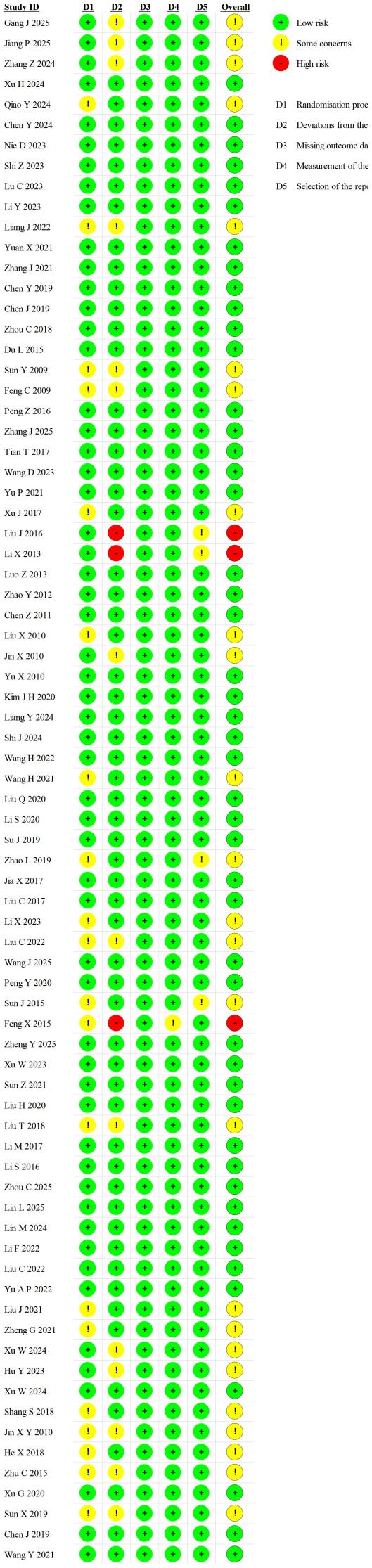


**Supplementary Figure 2** Bias risk plot.


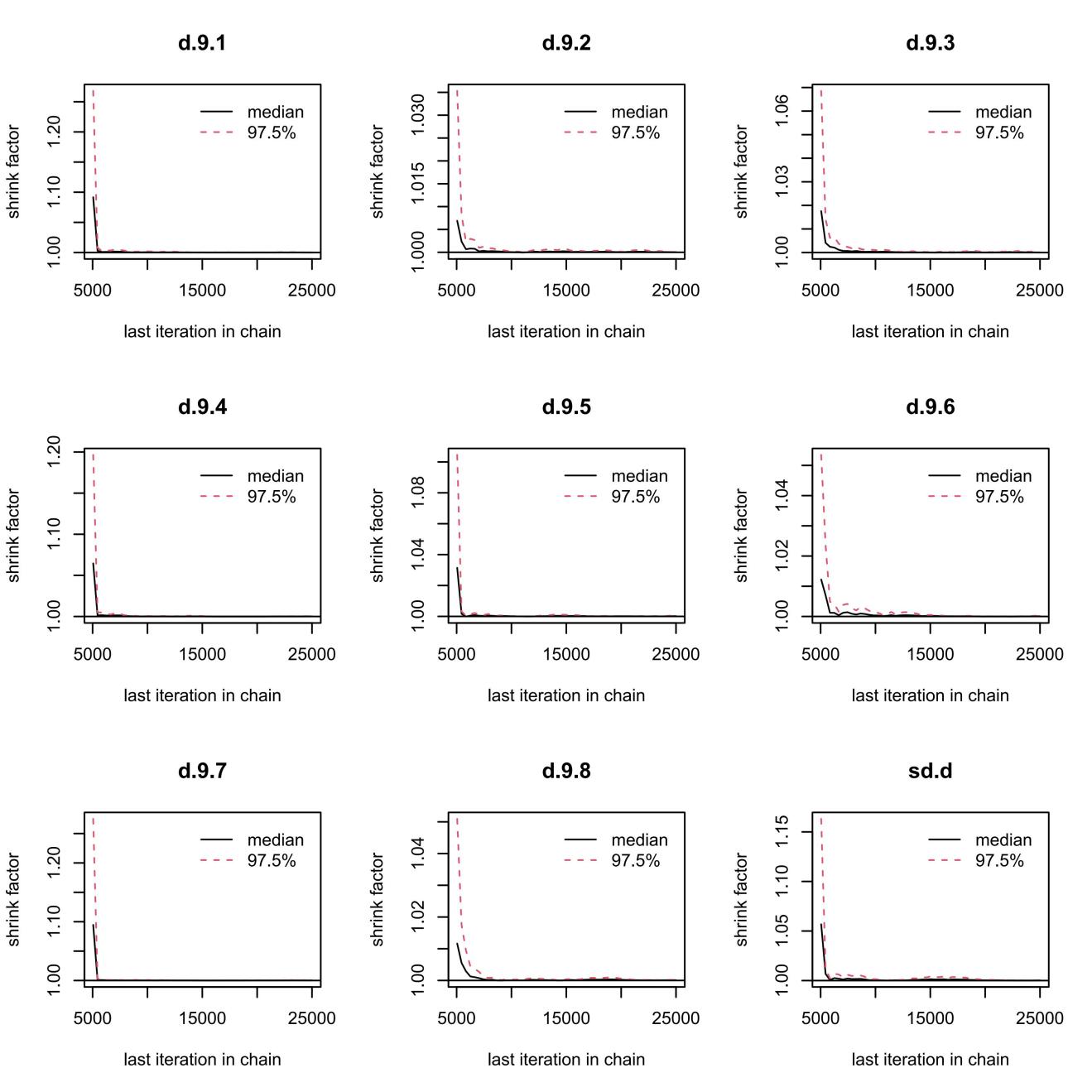


**Supplementary Figure 3** Convergence diagnostic plot for MMSE.


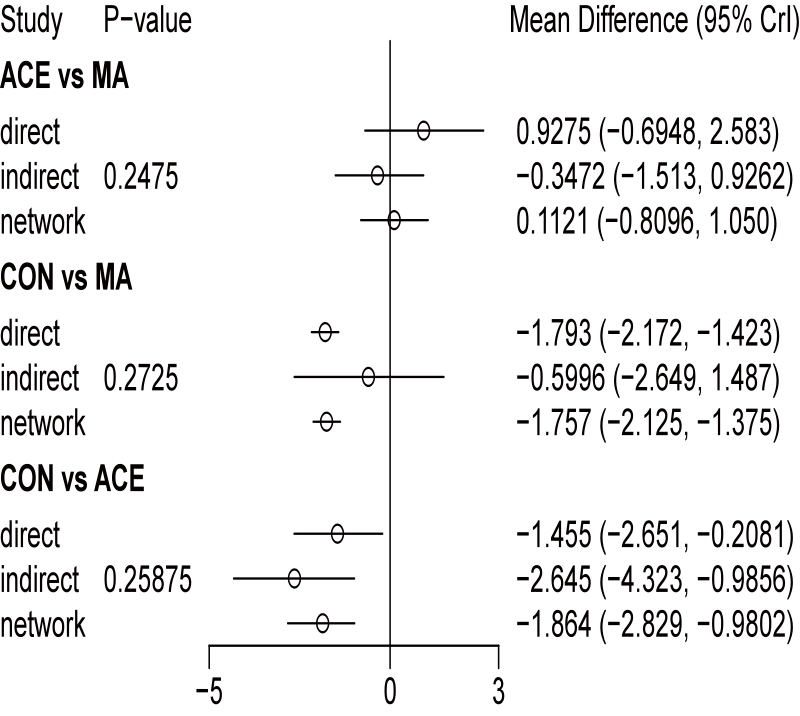


**Supplementary Figure 4** Inconsistency assessment plot.


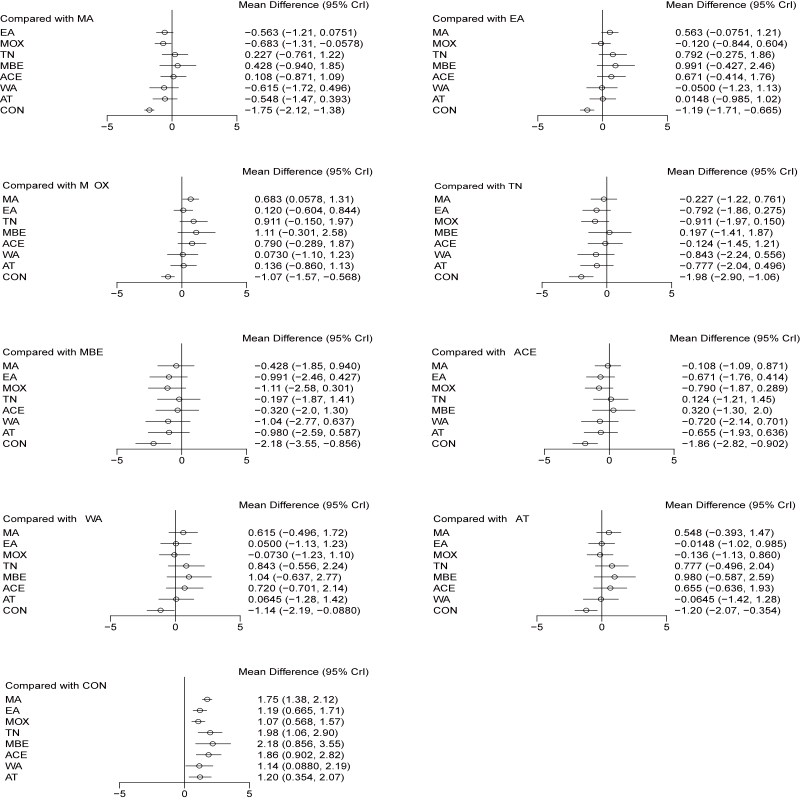


**Supplementary Figure 5** Forest plot of the NMA for MMSE scores.


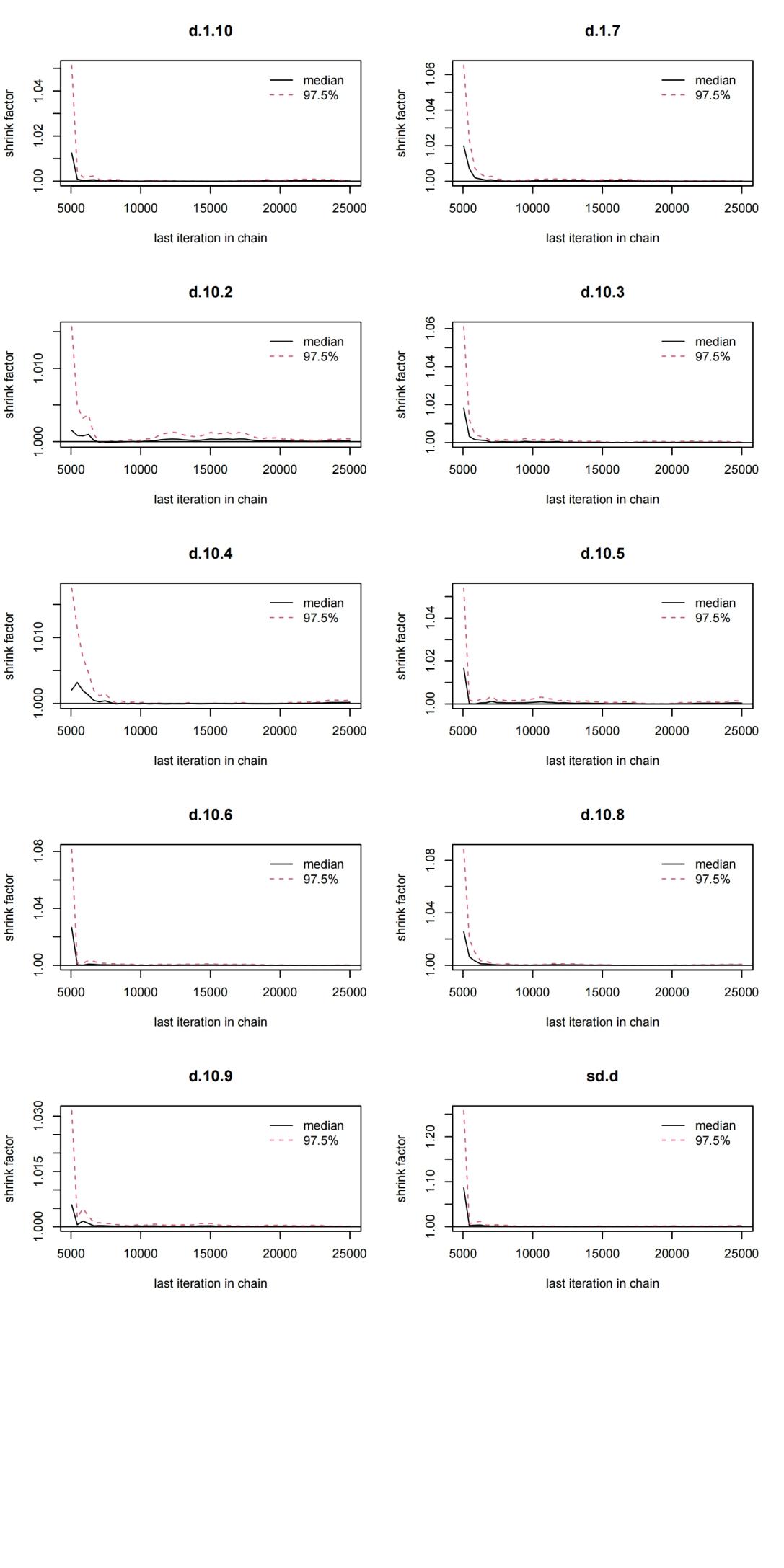


**Supplementary Figure 6** Convergence diagnostic plot for MoCA.


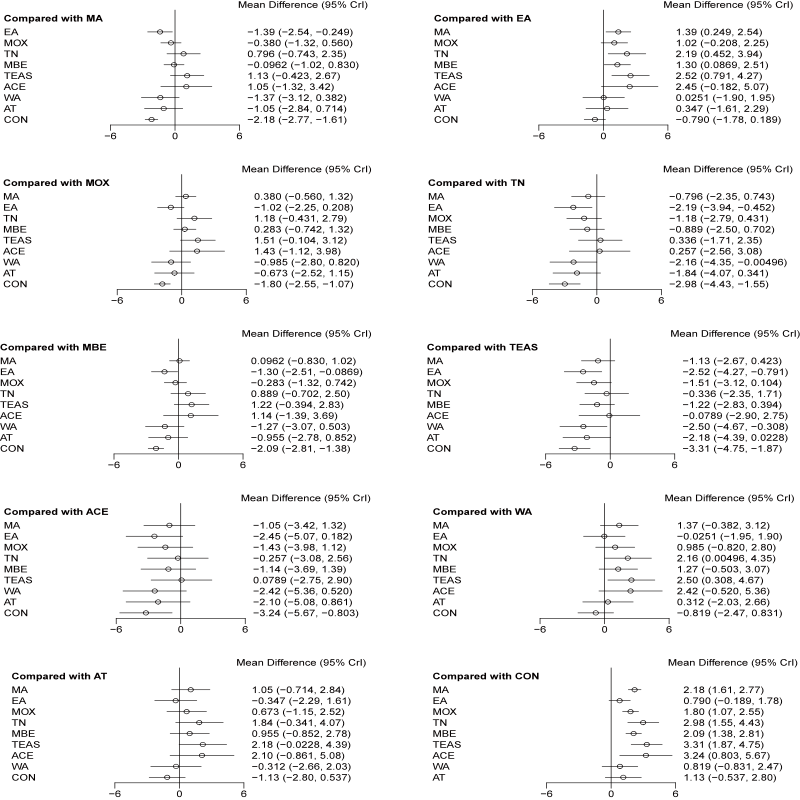


**Supplementary Figure 7** Forest plot of the NMA for MoCA scores.


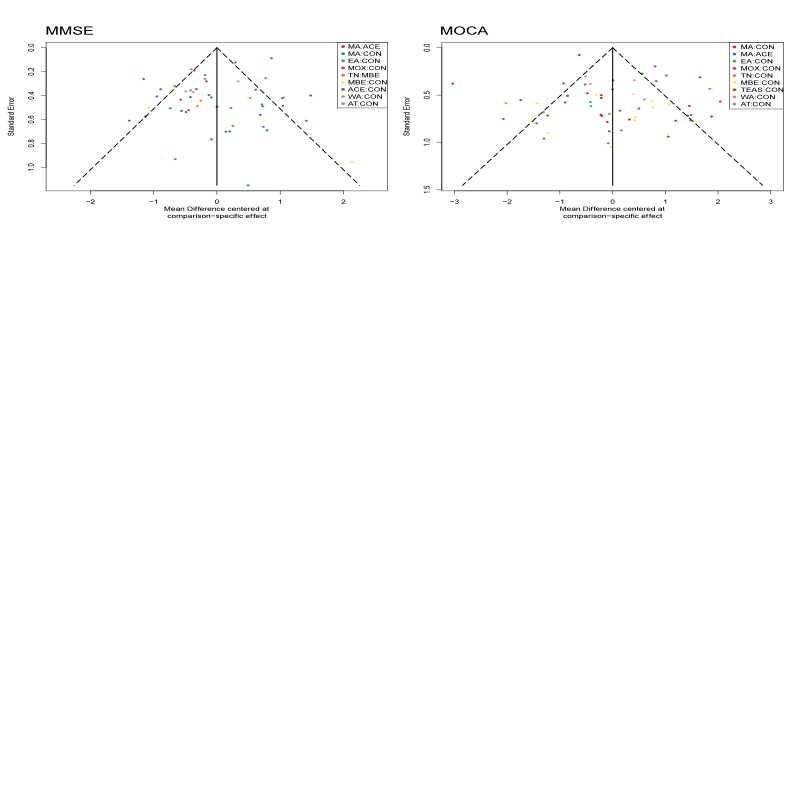


**Supplementary Figure 8** Funnel plots for publication bias assessment.


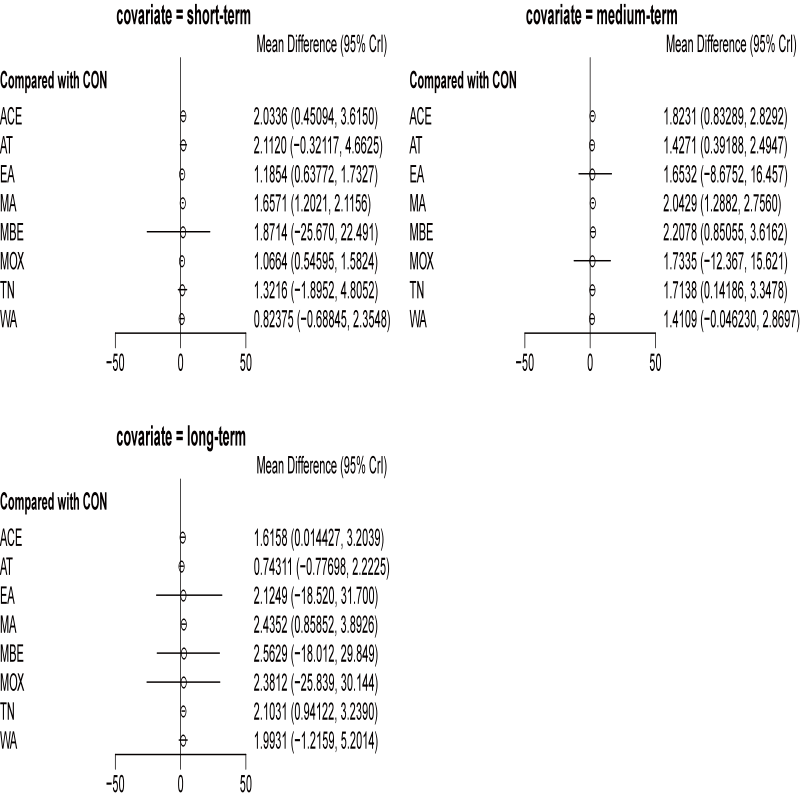


**Supplementary Figure 9** MMSE subgroup forest plot by intervention duration.


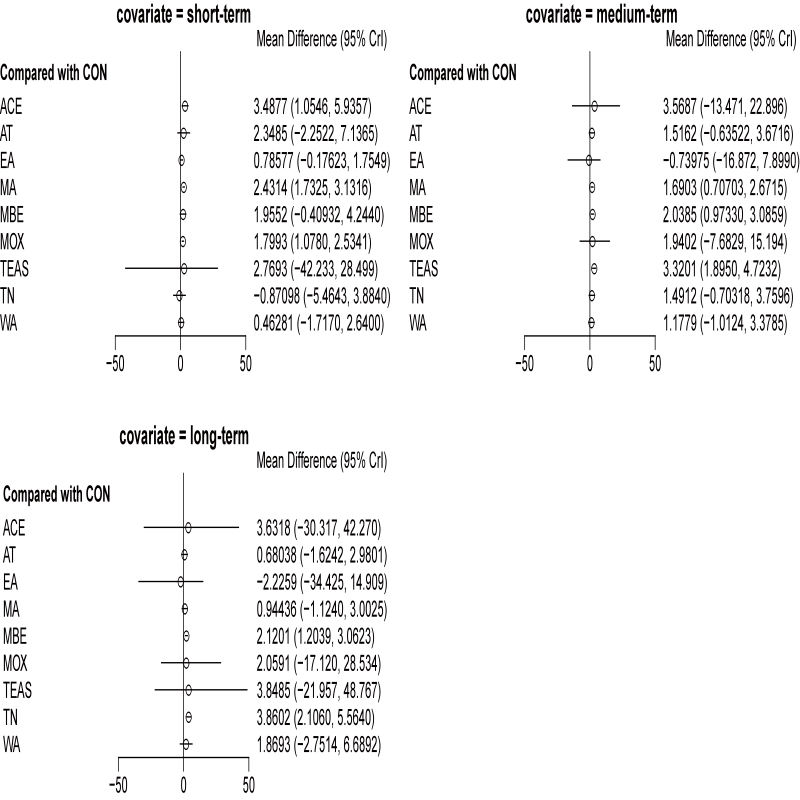


**Supplementary Figure 10** MoCA subgroup forest plot by intervention duration.


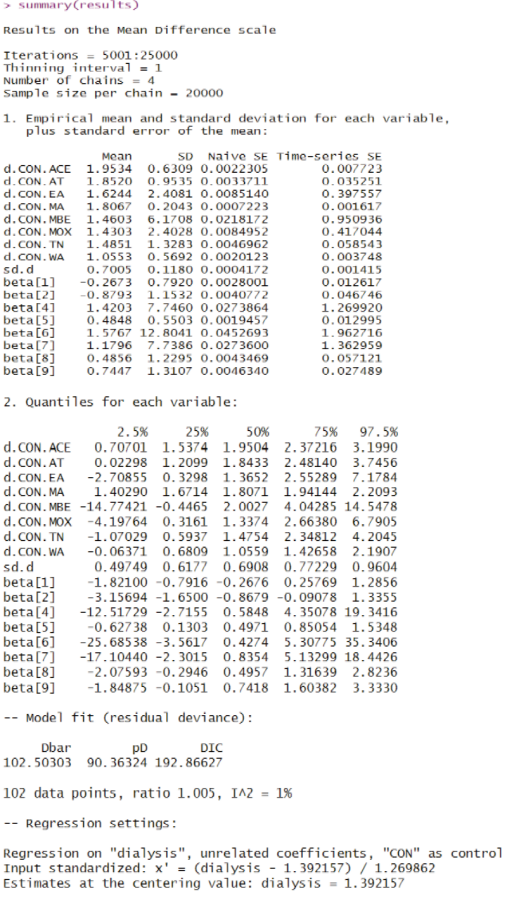


**Supplementary Figure 11** Meta-regression of intervention duration on MMSE scores.


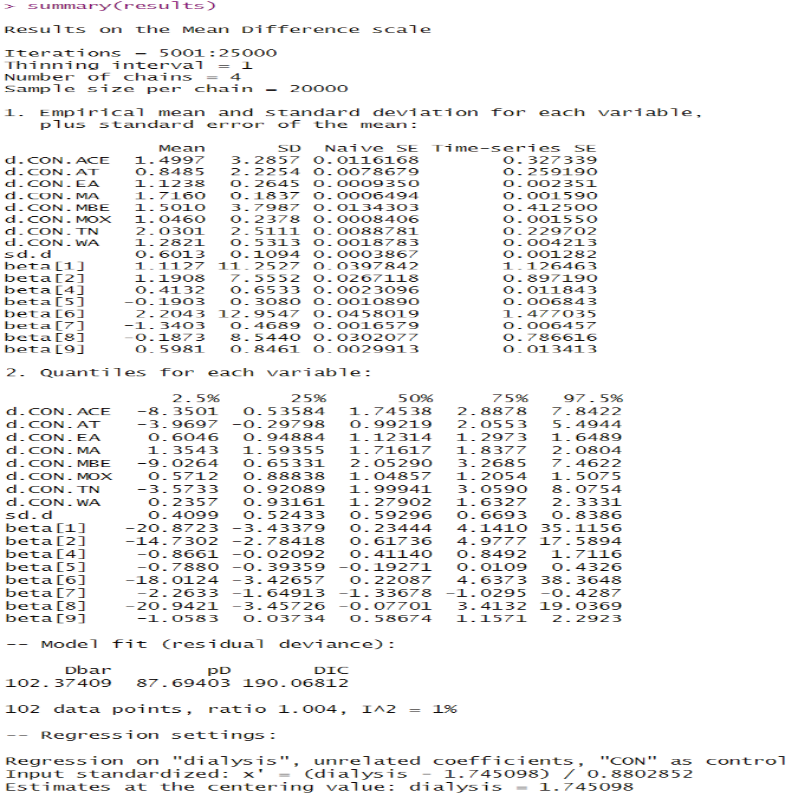


**Supplementary Figure 12** Meta-regression of intervention duration on MoCA scores.


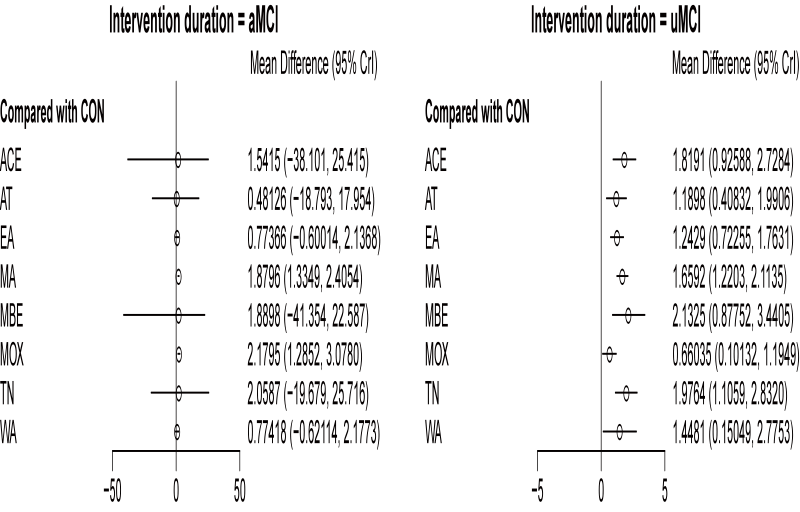


**Supplementary Figure 13** MMSE subgroup forest plot by intervention population.


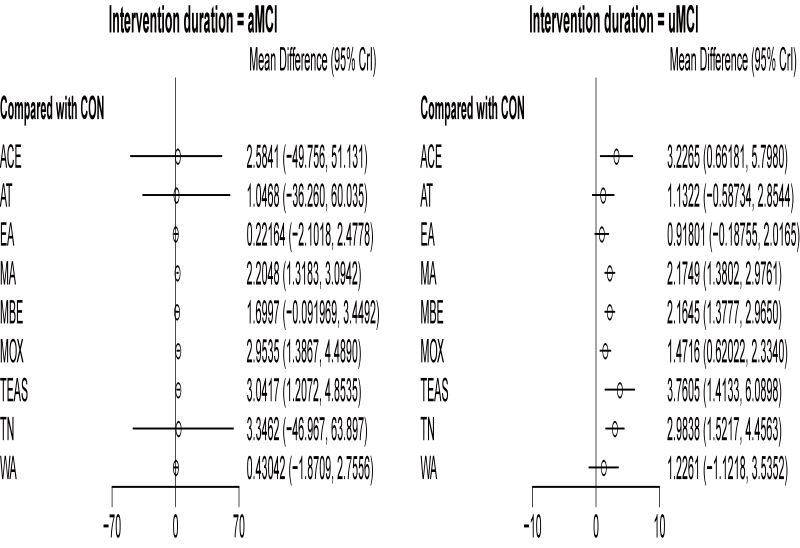


**Supplementary Figure 14** MoCA subgroup forest plot by intervention population.


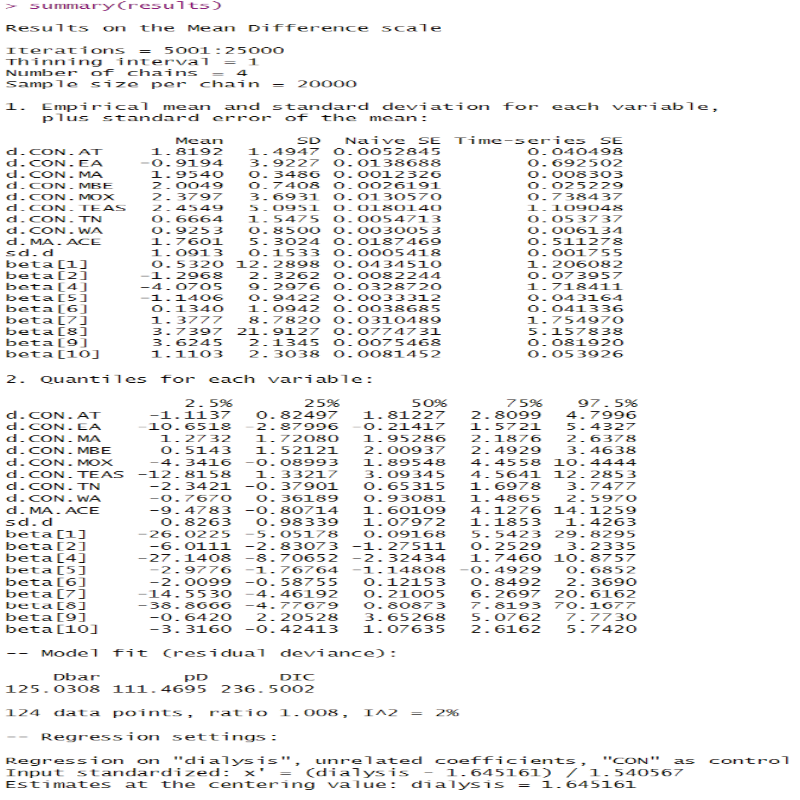


**Supplementary Figure 15** Meta-regression of intervention population on MMSE scores.


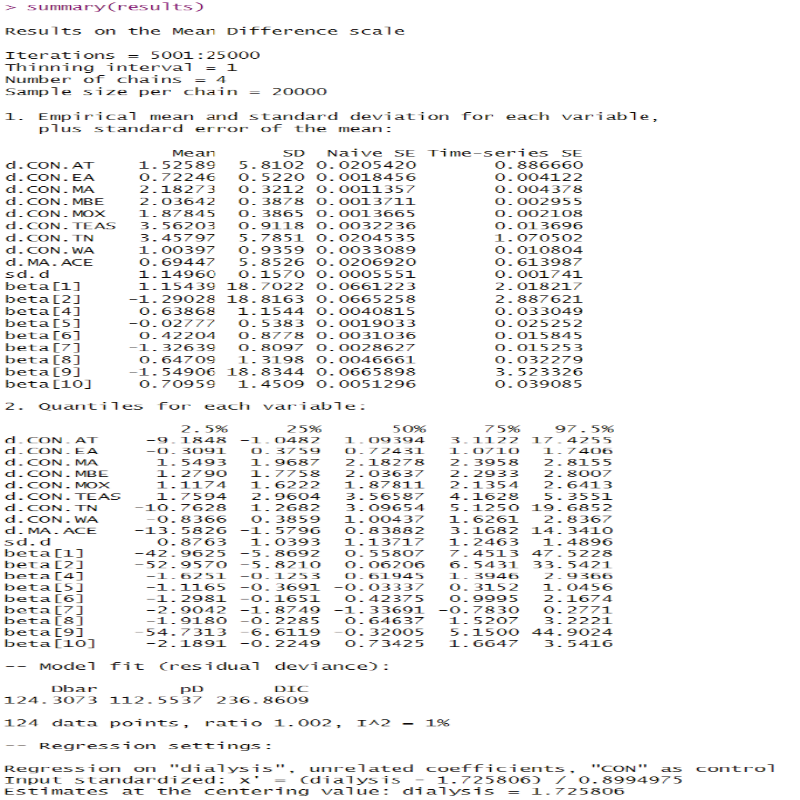


**Supplementary Figure 16** Meta-regression of intervention population on MoCA scores.


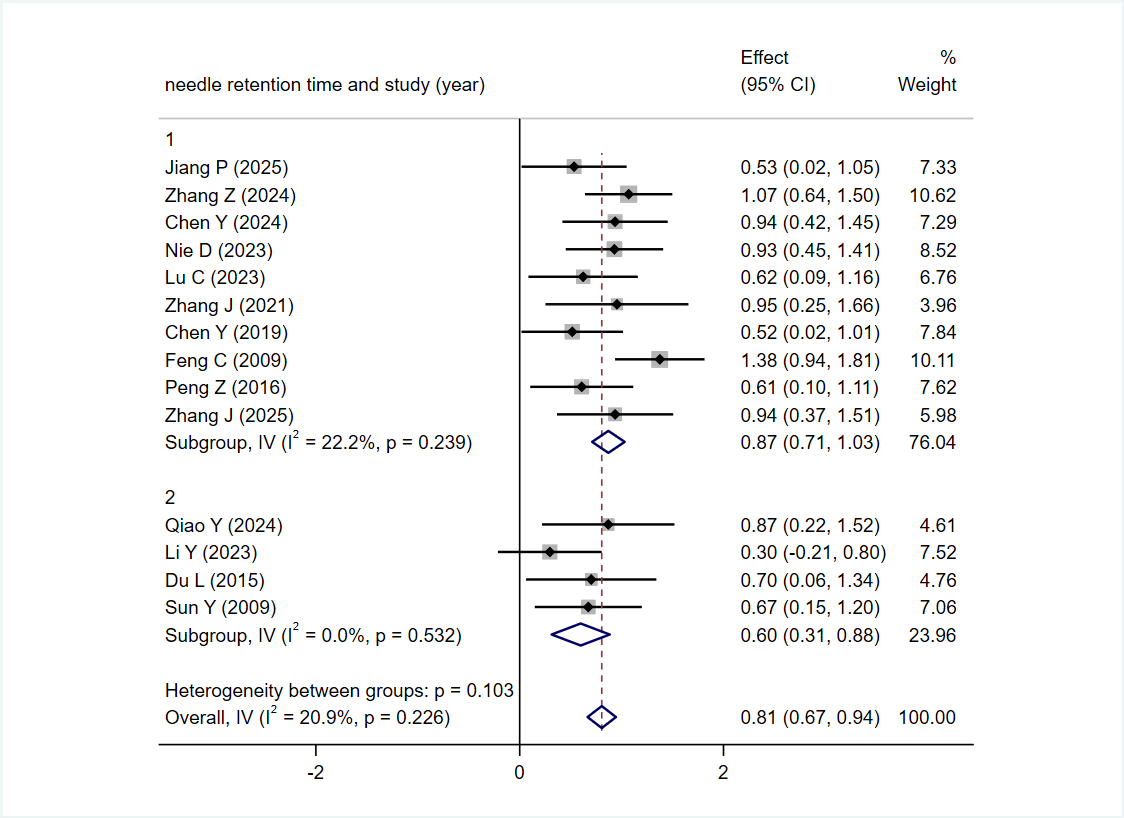


**Supplementary Figure 17** Acupuncture needle retention time subgroup analysis on MMSE scores. Only studies that explicitly reported acupuncture needle retention time were included, ensuring that at least two studies were used for the analysis. In the figure, 1 represents 30 minutes, and 2 represents 40 minutes.


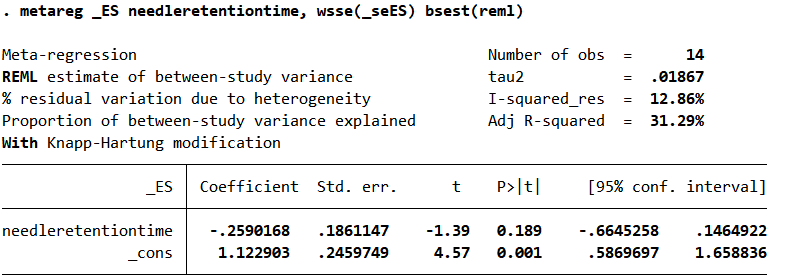


**Supplementary Figure 18 Meta-regression of acupuncture needle retention time on MMSE scores.**


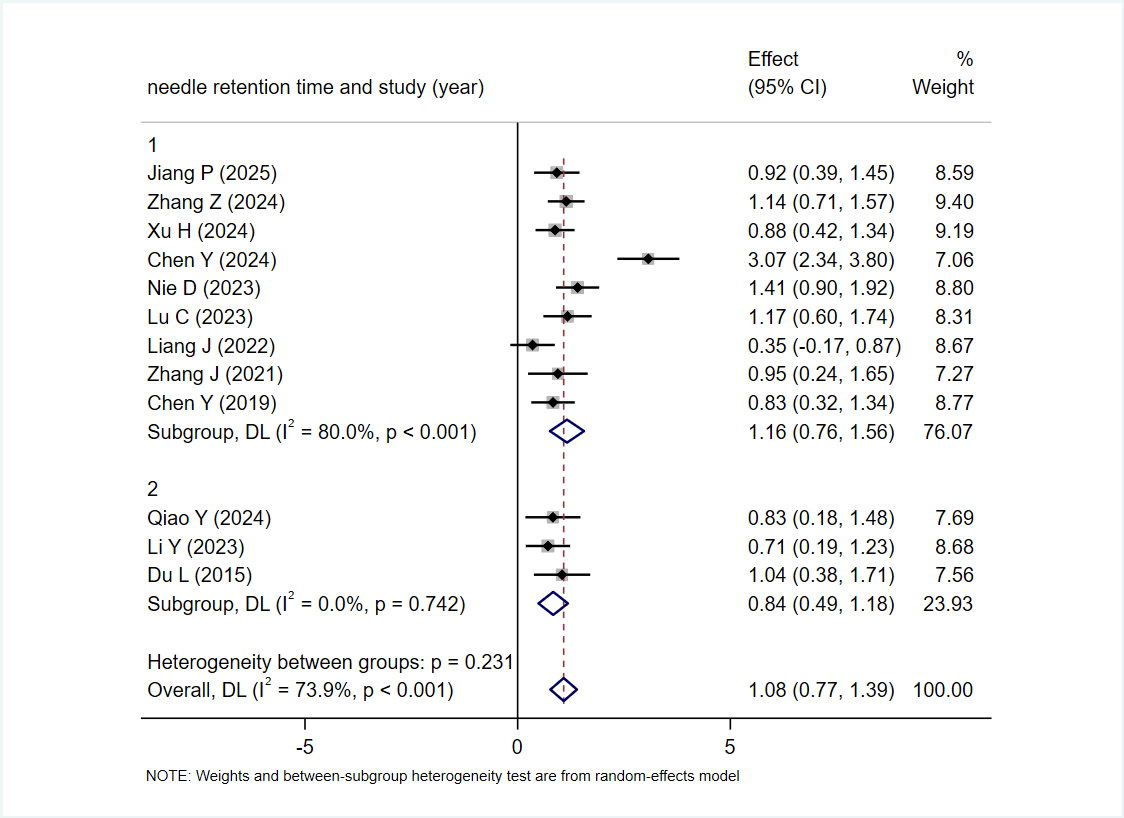


**Supplementary Figure 19** Acupuncture needle retention time subgroup analysis on MoCA scores. Only studies that explicitly reported acupuncture needle retention time were included, ensuring that at least two studies were used for the analysis. In the figure, 1 represents 30 minutes, and 2 represents 40 minutes.


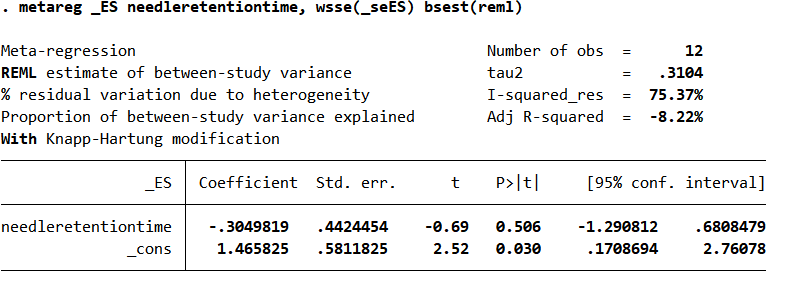


**Supplementary Figure 20 Meta-regression of acupuncture needle retention time on MoCA scores.**


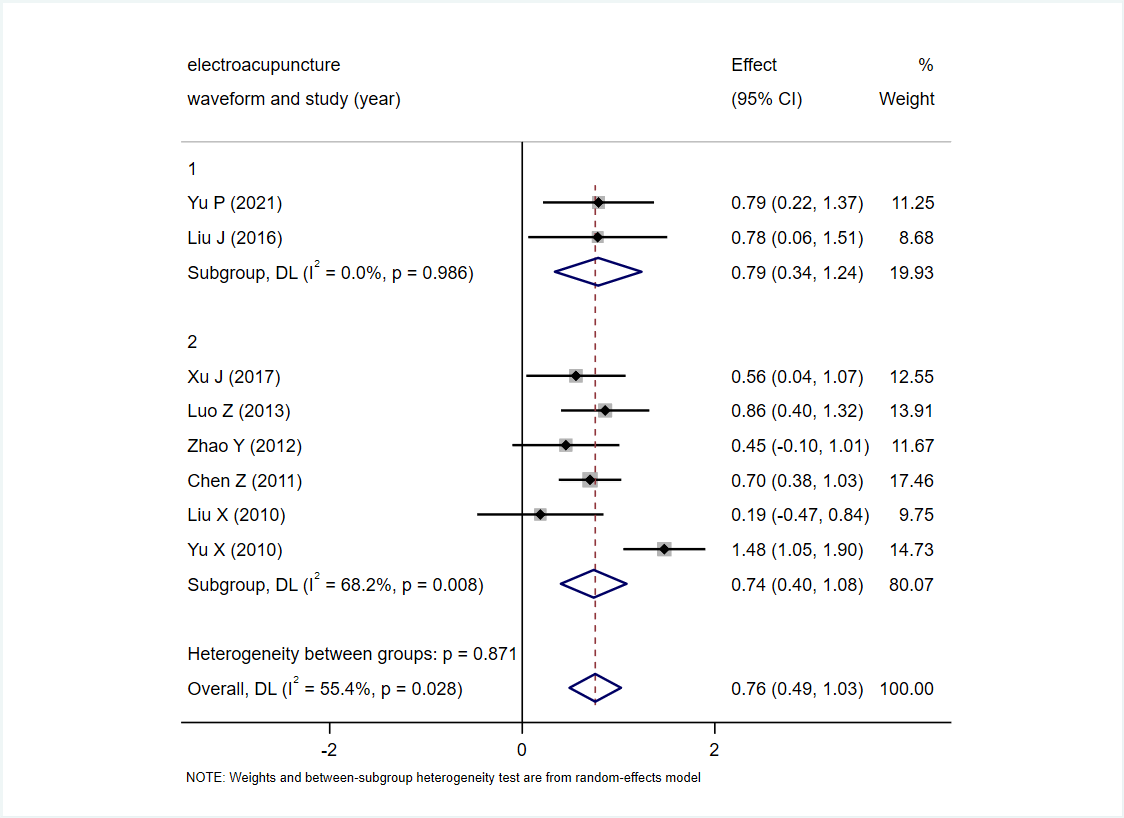


**Supplementary Figure 21** Electroacupuncture waveform subgroup analysis on MMSE scores. Only studies that explicitly reported electroacupuncture waveform were included, ensuring that at least two studies were used for the analysis. In the figure, 1 represents sparse-dense wave, and 2 represents continuous wave.


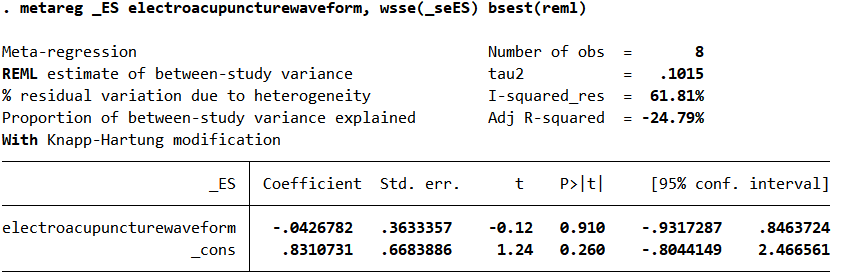


**Supplementary Figure 22 Meta-regression of electroacupuncture waveform on MMSE scores.**


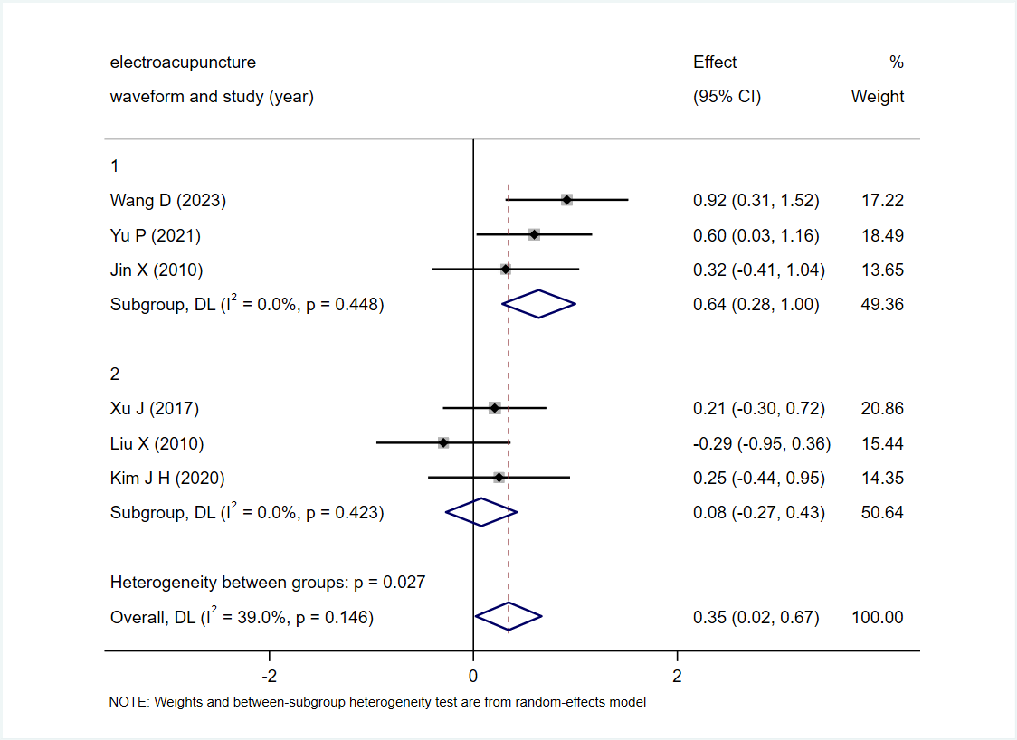


**Supplementary Figure 23 Electroacupuncture waveform subgroup analysis on MoCA scores.** Only studies that explicitly reported electroacupuncture waveform were included, ensuring that at least two studies were used for the analysis. In the figure, 1 represents sparse-dense wave, and 2 represents continuous wave.


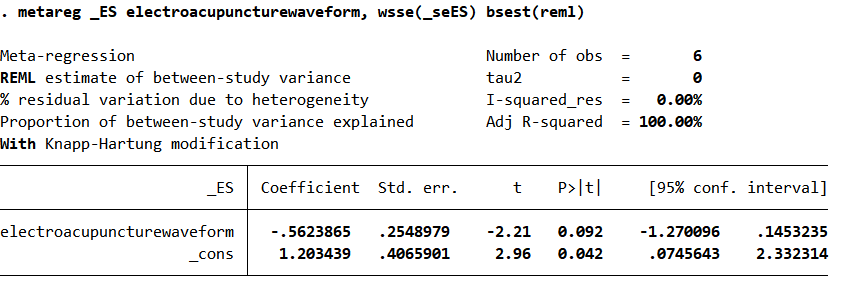


**Supplementary Figure 24 Meta-regression of electroacupuncture waveform on MoCA scores.**


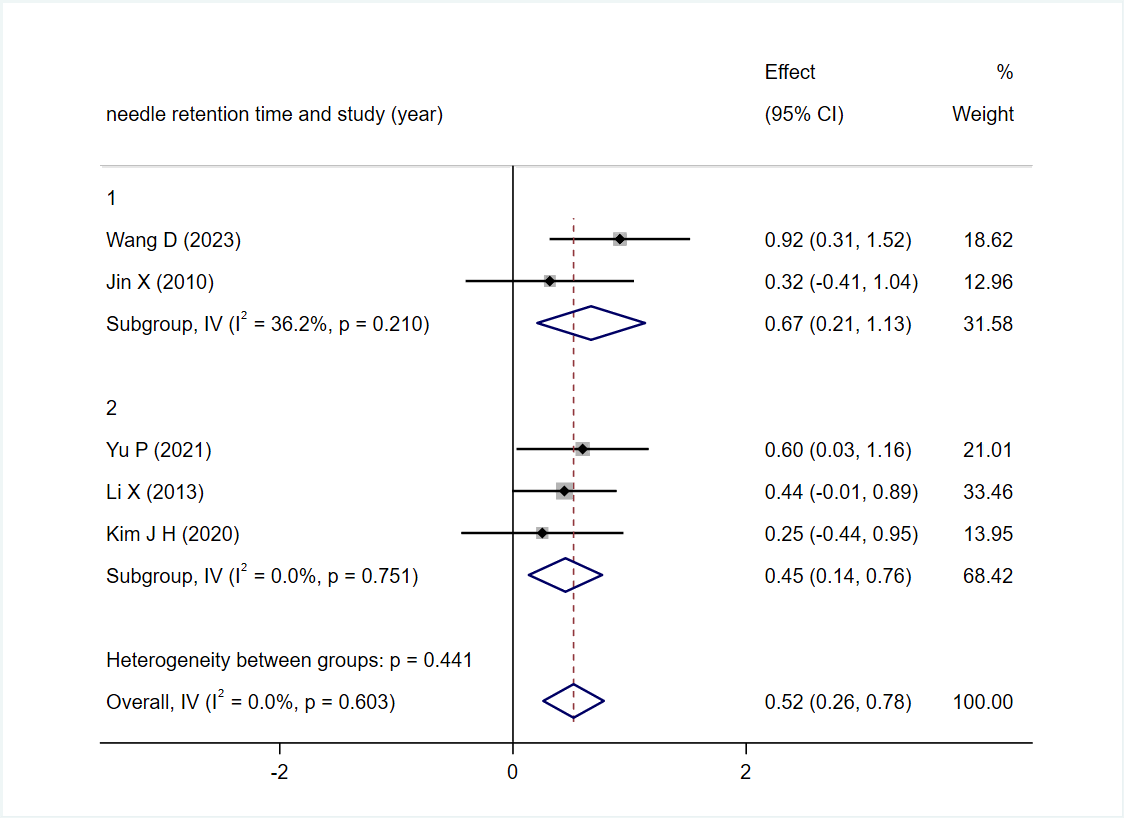


**Supplementary Figure 25** Electroacupuncture needle retention time subgroup analysis on MoCA scores. Only studies that explicitly reported electroacupuncture needle retention time were included, ensuring that at least two studies were used for the analysis. In the figure, 1 represents 20 minutes, and 2 represents 30 minutes.


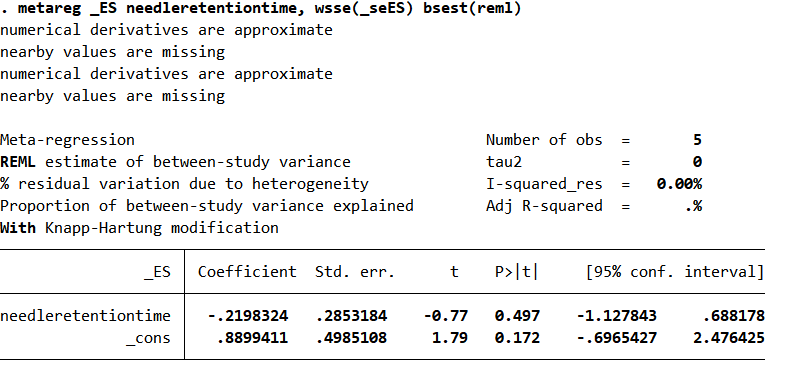


**Supplementary Figure 26 Meta-regression of electroacupuncture needle retention time on MoCA Scores.**


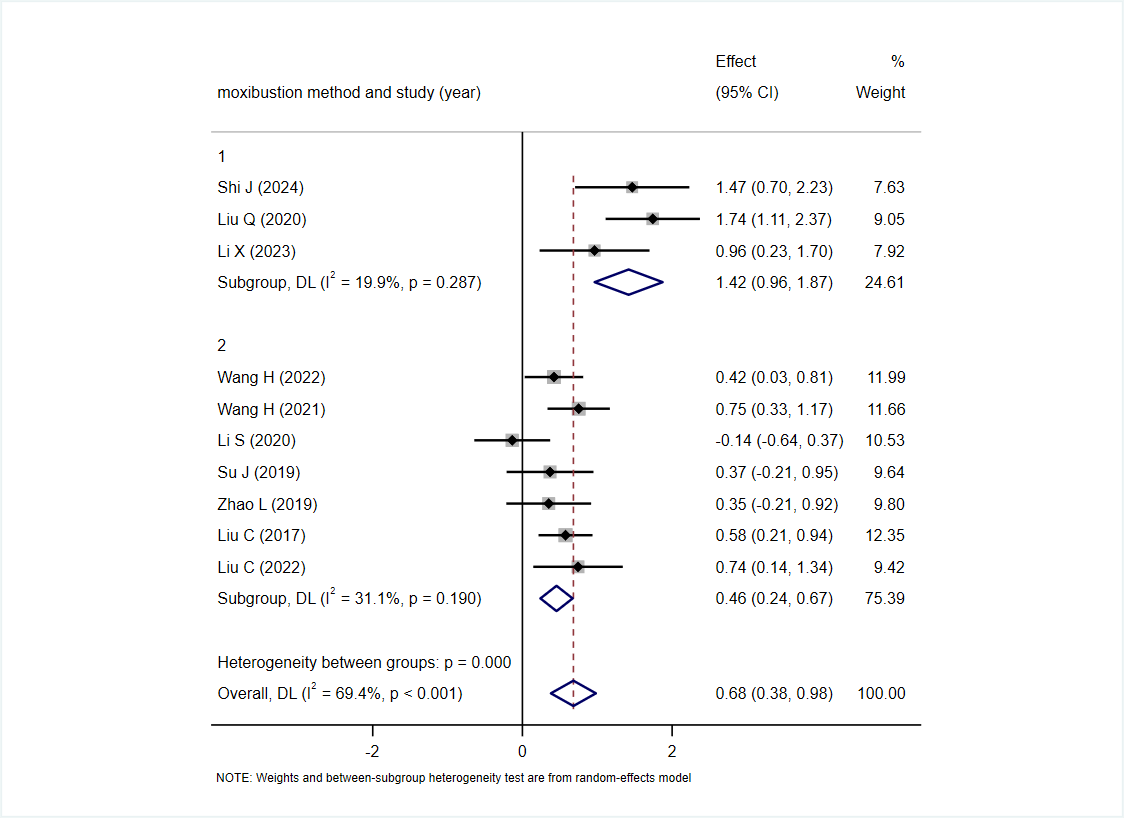


**Supplementary Figure 27 Moxibustion method subgroup analysis on MMSE scores.** In the figure, 1 represents moxibustion devices, and 2 represents traditional ignited moxibustion.


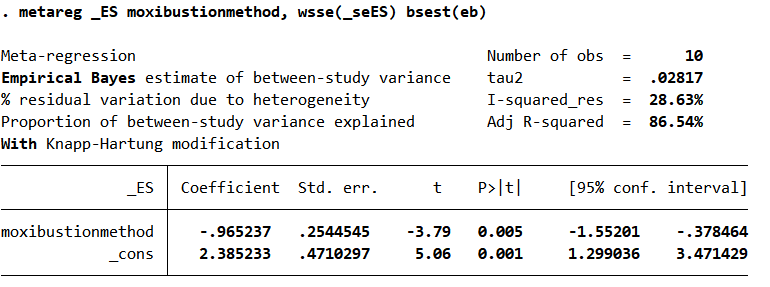


**Supplementary Figure 28** Meta-regression of MMSE scores by moxibustion method.


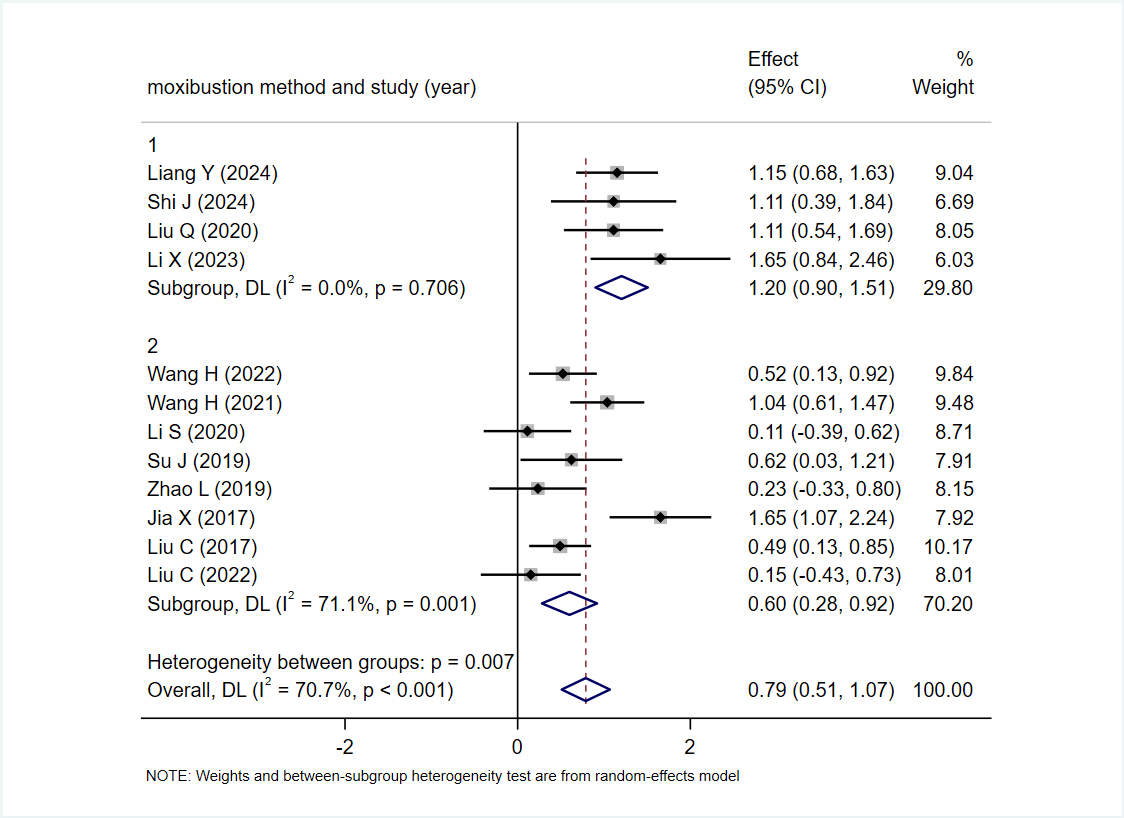


**Supplementary Figure 29** **Moxibustion method subgroup analysis on MOCA scores.**


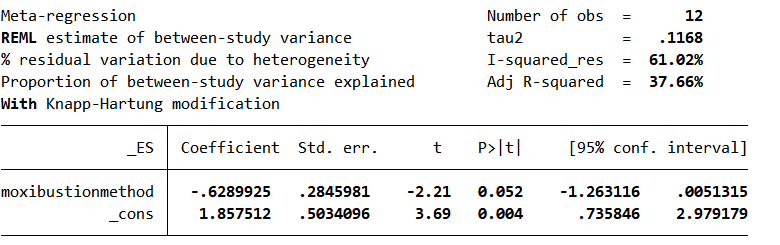


**Supplementary Figure 30** Meta-regression of MoCA scores by moxibustion method.


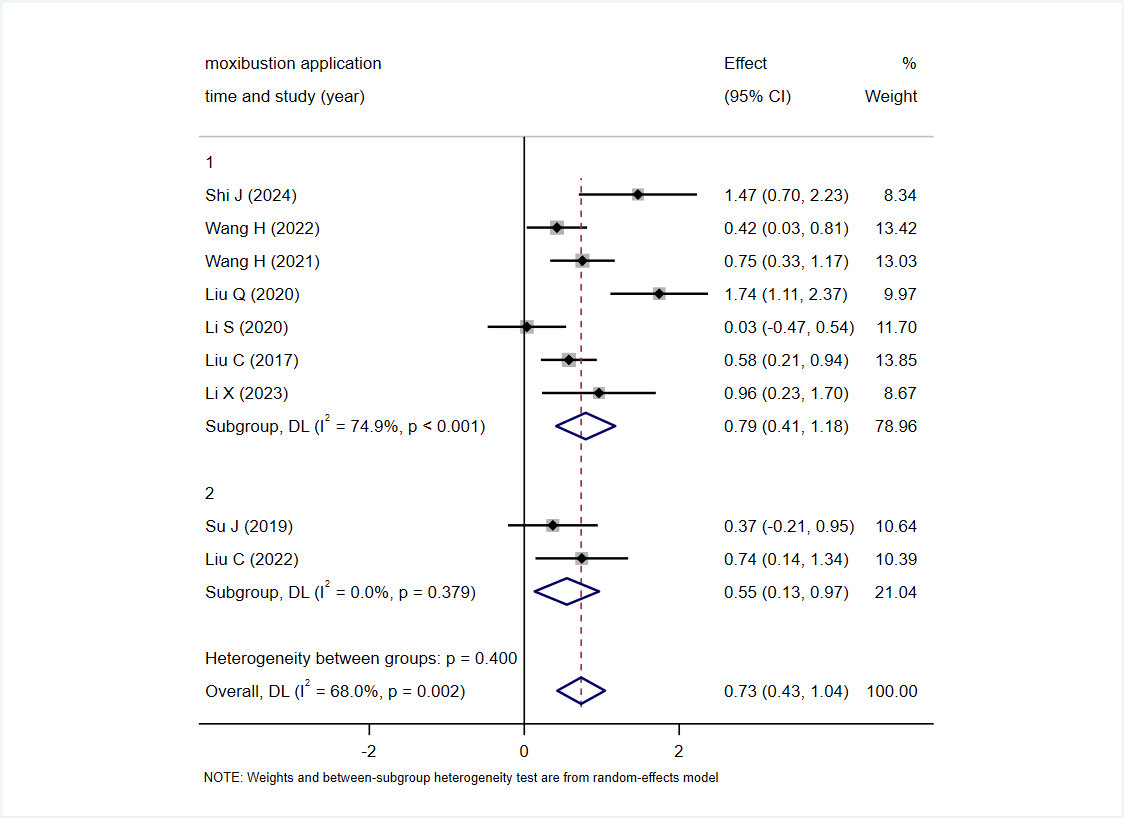


**Supplementary Figure 31** Moxibustion duration subgroup analysis on MMSE scores. Only studies that explicitly reported moxibustion duration were included, ensuring that at least two studies were used for the analysis. In the figure, 1 represents 20 minutes, and 2 represents studies that consistently describe moxibustion with 3 cones, lasting 10-15 minutes.


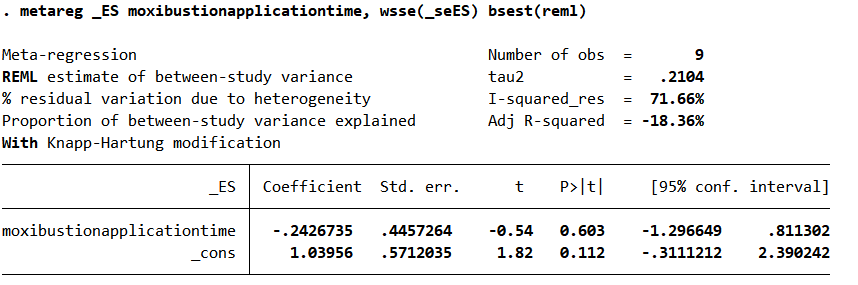


**Supplementary Figure 32** Meta-regression of moxibustion duration on MMSE scores.


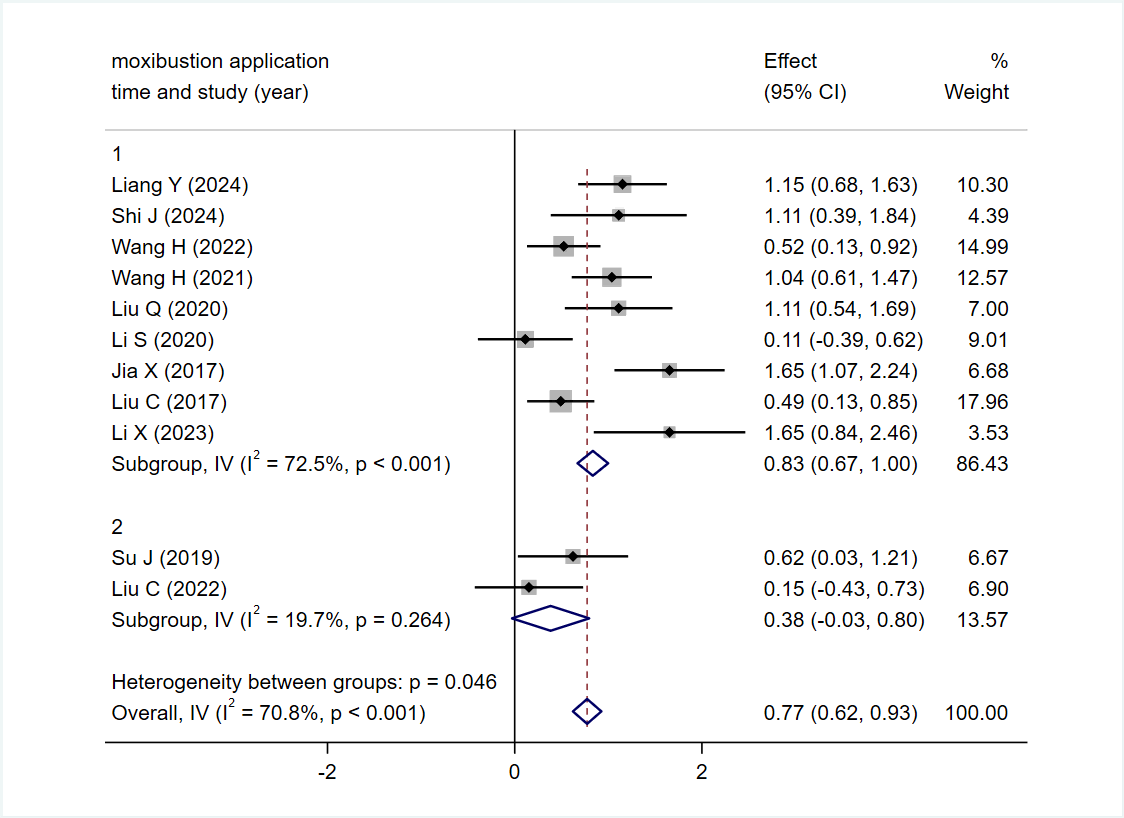


**Supplementary Figure 33** Moxibustion duration subgroup analysis on MoCA scores. Only studies that explicitly reported moxibustion duration were included, ensuring that at least two studies were used for the analysis. In the figure, 1 represents 20 minutes, and 2 represents studies that consistently describe moxibustion with 3 cones, lasting 10-15 minutes.


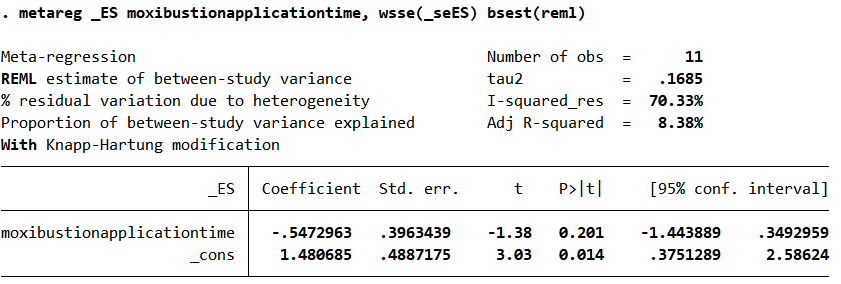


**Supplementary Figure 34 Meta-regression of moxibustion duration on MoCA scores.**


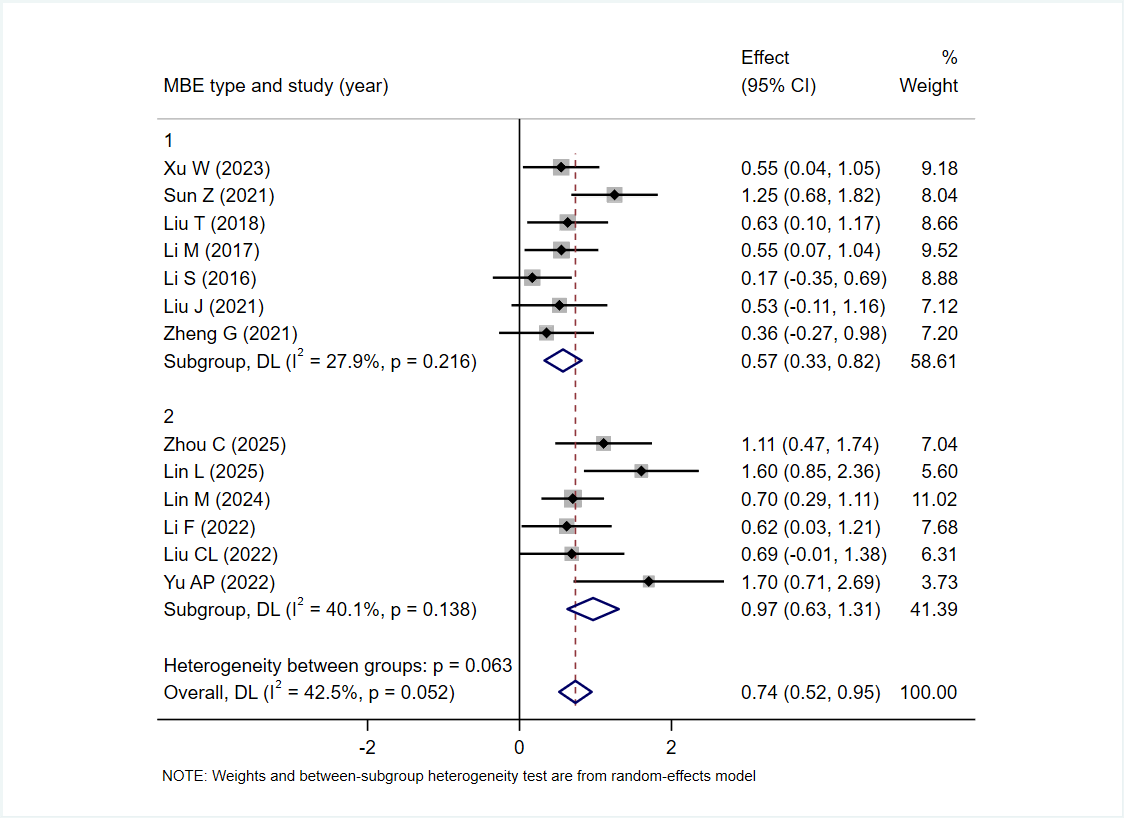


**Supplementary Figure 35** MBE type subgroup analysis on MoCA scores. Only studies that included at least two papers of the same MBE exercise type were included. In the figure, 1 represents Baduanjin, and 2 represents Tai Chi.


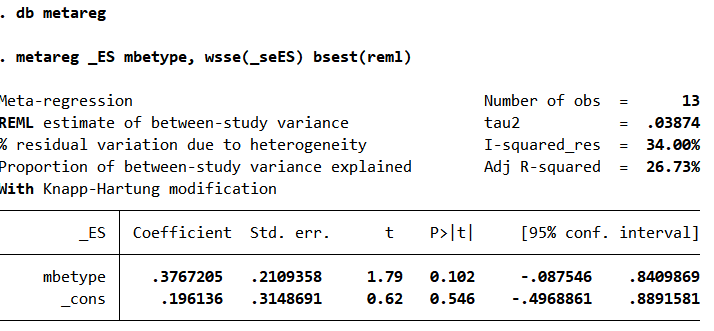


**Supplementary Figure 36** Meta-regression of MBE type on MoCA scores.

## **1.2 Supplementary Table**

# **Supplementary** Table 1 Search strategy.

## **Supplementary Table 1.1** Search strategy of PubMed.

| # | Searches |
| --- | --- |
| 1 | "Cognitive Dysfunction"[Mesh] |
| 2 | (((((Mild Cognitive Impairment[Title/Abstract]) OR (Cognitive Decline[Title/Abstract])) OR (Cognitive Dysfunctions[Title/Abstract])) OR (Cognitive Disorder[Title/Abstract])) OR (Mental Deterioration[Title/Abstract])) OR (Cognition Disorders[Title/Abstract]) |
| 3 | #1 OR #2 |
| 4 | "Randomized Controlled Trials as Topic"[Mesh] |
| 5 | ((((((((Randomized controlled trial[Title/Abstract]) OR (Controlled clinical trial[Title/Abstract])) OR (Randomized[Title/Abstract])) OR (Placebo[Title/Abstract])) OR (Randomly[Title/Abstract])) OR (Random[Title/Abstract])) OR (Controlled Clinical Trials, Randomized[Title/Abstract])) OR (Clinical Trials, Randomized[Title/Abstract])) OR (Random*[Title/Abstract]) |
| 6 | #4 OR #5 |
| 7 | (((((((((((("Medicine, Chinese Traditional"[Mesh]) OR (((Traditional Chinese Medicine[Title/Abstract]) OR (Chinese Traditional Medicine[Title/Abstract])) OR (Traditional Chinese non-pharmacological therapy[Title/Abstract]))) OR ("Acupuncture"[Mesh])) OR (((Acupuncture Treatment[Title/Abstract]) OR (Acupuncture therapy[Title/Abstract])) OR (Acupuncture stimulation[Title/Abstract]))) OR ("Electroacupuncture"[Mesh])) OR (((Electrical acupuncture[Title/Abstract]) OR (Electroacupuncture therapy[Title/Abstract])) OR (Electrical needle therapy[Title/Abstract]))) OR ("Moxibustion"[Mesh])) OR (((Moxibustion therapy[Title/Abstract]) OR (Moxa therapy[Title/Abstract])) OR (Moxa treatment[Title/Abstract]))) OR ("Massage"[Mesh])) OR (((Massage Therapy[Title/Abstract]) OR (Tuina[Title/Abstract])) OR (Zone Therapy[Title/Abstract]))) OR (((((((((((Acupoint catgut embedding[Title/Abstract]) OR (Acupoint thread-embedding[Title/Abstract])) OR (Acupoint Threading[Title/Abstract])) OR (Warm Acupuncture[Title/Abstract])) OR (Moxibustion Acupuncture[Title/Abstract])) OR (Warming Needle Moxibustion[Title/Abstract])) OR (Warm needle therapy[Title/Abstract])) OR (Auricular Therapy[Title/Abstract])) OR (Ear acupuncture[Title/Abstract])) OR (Ear point stimulation[Title/Abstract])) OR (Auricular acupuncture[Title/Abstract]))) OR ((("Qigong"[Mesh]) OR "Tai Ji"[Mesh]) OR "Mind-Body Therapies"[Mesh])) OR ((((((Baduanjin[Title/Abstract]) OR (Yijinjing[Title/Abstract])) OR (Ch'i Kung[Title/Abstract])) OR (Tai Ji Quan[Title/Abstract])) OR (Tai Chi[Title/Abstract])) OR (Mind-Body[Title/Abstract])) |
| 7 | #3 AND #6 AND #7 |

## **Supplementary Table 1.2** Search strategy of Web of Science.

| # | Searches |
| --- | --- |
| 1 | ((((((TS=(Cognitive Dysfunction)) OR TS=(Mild Cognitive Impairment)) OR TS=(Cognitive Decline)) OR TS=(Cognitive Dysfunctions)) OR TS=(Cognitive Disorder)) OR TS=(Mental Deterioration)) OR TS=(Cognition Disorders) |
| 2 | (((((((TS=(Random*)) OR TS=("Randomized controlled trial")) OR TS=("Controlled clinical trial")) OR TS=(Randomized*)) OR TS=(Placebo*)) OR TS=(Randomly)) OR TS=("Controlled Clinical Trials, Randomized")) OR TS=("Clinical Trials, Randomized") |
| 3 | ((((((((((((((((((((((((((((((((((((((((TS=(Medicine, Chinese Traditional)) OR TS=(Traditional Chinese Medicine)) OR TS=(Chinese Traditional Medicine)) OR TS=(Traditional Chinese non-pharmacological therapy)) OR TS=(Acupuncture)) OR TS=(Acupuncture Treatment)) OR TS=(Acupuncture therapy)) OR TS=(Acupuncture stimulation)) OR TS=(Electroacupuncture)) OR TS=(Electrical acupuncture)) OR TS=(Electroacupuncture therapy)) OR TS=(Electrical needle therapy)) OR TS=(Moxibustion)) OR TS=(Moxibustion therapy)) OR TS=(Moxa therapy)) OR TS=(Moxa treatment)) OR TS=(Massage)) OR TS=(Massage Therapy)) OR TS=(Tuina)) OR TS=(Zone Therapy)) OR TS=(Acupoint catgut embedding)) OR TS=(Acupoint thread-embedding)) OR TS=(Acupoint Threading)) OR TS=(Warm Acupuncture)) OR TS=(Moxibustion Acupuncture)) OR TS=(Warming Needle Moxibustion)) OR TS=(Warm needle therapy)) OR TS=(Auricular Therapy)) OR TS=(Ear acupuncture)) OR TS=(Ear point stimulation)) OR TS=(Auricular acupuncture)) OR TS=(Qigong)) OR TS=(Tai Ji))) OR TS=(Mind-Body Therapies)) OR TS=(Baduanjin)) OR TS=(Yijinjing)) OR TS=(Ch'i Kung)) OR TS=(Tai Ji Quan)) OR TS=(Tai Chi)) OR TS=(Mind-Body) |
| 4 | #1 AND #2 AND #3 |

## **Supplementary Table 1.3** Search strategy of Cochrane Library.

| # | Searches |
| --- | --- |
| 1 | MeSH descriptor: [Cognitive Dysfunction] explode all trees |
| 2 | (Cognitive Dysfunction OR Mild Cognitive Impairment OR Cognitive Decline OR Cognitive Dysfunctions OR Cognitive Disorder OR Mental Deterioration OR Cognition Disorders):ti,ab,kw |
| 3 | #1 OR #2 |
| 4 | MeSH descriptor: [Randomized Controlled Trials as Topic] explode all trees |
| 5 | (Random* OR Randomized controlled trial OR Controlled clinical trial OR Randomized OR Placebo OR Randomly OR Controlled Clinical Trials, Randomized OR Clinical Trials, Randomized):ti,ab,kw |
| 6 | #4 OR #5 |
| 7 | MeSH descriptor: [Medicine, Chinese Traditional] explode all trees |
| 8 | MeSH descriptor: [Acupuncture] explode all trees |
| 9 | MeSH descriptor: [Electroacupuncture] explode all trees |
| 10 | MeSH descriptor: [Moxibustion] explode all trees |
| 11 | MeSH descriptor: [Massage] explode all trees |
| 12 | MeSH descriptor: [Qigong] explode all trees |
| 13 | MeSH descriptor: [Tai Ji] explode all trees |
| 14 | MeSH descriptor: [Mind-Body Therapies] explode all trees |
| 15 | (Acupuncture Treatment OR Acupuncture therapy acupuncture stimulation OR electrical acupuncture OR electroacupuncture therapy OR electrical needle therapy OR moxibustion therapy OR moxa therapy OR moxa treatment OR Massage Therapy OR tuina OR Zone Therapy OR acupoint catgut embedding OR acupoint thread-embedding OR Acupoint Threading OR Warm Acupuncture OR Moxibustion Acupuncture OR Warming Needle Moxibustion OR Warm needle therapy OR Auricular Therapy OR Ear acupuncture OR Ear point stimulation OR Auricular acupuncture OR Baduanjin OR Yijinjing OR Ch'i Kung OR Tai Ji Quan OR Mind-Body OR Tai Chi):ti,ab,kw |
| 16 | #7 OR #8 OR #9 OR #10 OR #11 OR #12 OR #13 OR #14 OR #15 |
| 17 | #3 AND #6 AND #16 |

## **Supplementary Table 1.4** Search strategy of Embase.

| # | Searches |
| --- | --- |
| 1 | 'cognitive defect'/exp OR 'cognitive decline':ti,ab,kw OR 'cognitive dysfunctions':ti,ab,kw OR 'cognitive disorder':ti,ab,kw OR 'mental deterioration':ti,ab,kw OR 'cognition disorders':ti,ab,kw OR 'mild cognitive impairment'/exp OR 'amnestic mild cognitive impairment':ti,ab,kw OR 'mild cognitive impairment':ti,ab,kw |
| 2 | 'randomized controlled trial (topic)'/exp OR random*:ti,ab,kw OR 'randomized controlled trial':ti,ab,kw OR 'controlled clinical trial':ti,ab,kw OR randomized*:ti,ab,kw OR placebo*:ti,ab,kw OR randomly:ti,ab,kw OR 'controlled clinical trials, randomized':ti,ab,kw OR 'clinical trials, randomized':ti,ab,kw |
| 3 | 'medicine, chinese traditional':ti,ab,kw OR 'traditional chinese medicine':ti,ab,kw OR 'chinese traditional medicinet':ti,ab,kw OR 'traditional chinese non-pharmacological therapy':ti,ab,kw OR 'acupuncture'/exp OR 'acupuncture treatment':ti,ab,kw OR 'acupuncture therapy':ti,ab,kw OR 'acupuncture stimulation':ti,ab,kw OR 'electroacupuncture'/exp OR 'electrical acupuncture':ti,ab,kw OR 'electroacupuncture therapy':ti,ab,kw OR 'electrical needle therapy':ti,ab,kw OR 'moxibustion'/exp OR 'moxibustion therapy':ti,ab,kw OR 'moxa therapy':ti,ab,kw OR 'moxa treatment':ti,ab,kw OR 'massage'/exp OR 'massage therapy':ti,ab,kw OR 'tuina':ti,ab,kw OR 'zone therapy':ti,ab,kw OR 'acupoint catgut embedding':ti,ab,kw OR 'acupoint thread-embedding':ti,ab,kw OR 'acupoint threading':ti,ab,kw OR 'warm acupuncture'/exp OR 'moxibustion acupuncture':ti,ab,kw OR 'warming needle moxibustion':ti,ab,kw OR 'warm needle therapy':ti,ab,kw OR 'auricular therapy':ti,ab,kw OR 'ear acupuncture':ti,ab,kw OR 'ear point stimulation':ti,ab,kw OR 'auricular acupuncture':ti,ab,kw OR 'qigong'/exp OR 'tai ji'/exp OR 'mind-body therapies':ti,ab,kw OR baduanjin:ti,ab,kw OR yijinjing:ti,ab,kw OR 'chi kung':ti,ab,kw OR 'tai ji quan':ti,ab,kw OR 'tai chi':ti,ab,kw OR 'mind body':ti,ab,kw |
| 4 | #1 AND #2 AND #3 |

## **Supplementary Table 1.5** Search strategy of CNKI.

| # | Searches |
| --- | --- |
| 1 | （主题：针刺 + 针灸 + 电针+ 温针+ 艾灸 + 推拿 + 穴位+ 气功 + 八段锦 + 太极 + 易筋经） AND （主题：认知障碍 + 认知衰退 + 轻度认知障碍） |

## **Supplementary Table 1.6** Search strategy of Wanfang.

| # | Searches |
| --- | --- |
| 1 | [主题:(针刺 or 针灸 or 电针 or 温针 or 艾灸 or 推拿 or 穴位 or 气功 or 气功 or 易筋经 or 太极 or 八段锦) and 主题:(认知障碍 or 认知衰退 or 轻度认知障碍)](https://s.wanfangdata.com.cn/advanced-search/paper?q=%E4%B8%BB%E9%A2%98:(%E9%92%88%E5%88%BA or %E9%92%88%E7%81%B8 or %E7%94%B5%E9%92%88 or %E6%B8%A9%E9%92%88 or %E8%89%BE%E7%81%B8 or %E6%8E%A8%E6%8B%BF or %E7%A9%B4%E4%BD%8D or %E6%B0%94%E5%8A%9F or %E6%B0%94%E5%8A%9F or %E6%98%93%E7%AD%8B%E7%BB%8F or %E5%A4%AA%E6%9E%81 or %E5%85%AB%E6%AE%B5%E9%94%A6) and %E4%B8%BB%E9%A2%98:(%E8%AE%A4%E7%9F%A5%E9%9A%9C%E7%A2%8D or %E8%AE%A4%E7%9F%A5%E8%A1%B0%E9%80%80 or %E8%BD%BB%E5%BA%A6%E8%AE%A4%E7%9F%A5%E9%9A%9C%E7%A2%8D)&searchtype=expert&type=["periodical","thesis","conference"]&chineseEnglishExpand=true" \t "https://s.wanfangdata.com.cn/advanced-search/_blank) |

## **Supplementary Table 1.7** Search strategy of VIP.

| # | Searches |
| --- | --- |
| 1 | [(((((((((((题名或关键词=针刺 OR 题名或关键词=针灸) OR 题名或关键词=电针) OR 题名或关键词=温针) OR 题名或关键词=艾灸) OR 题名或关键词=穴位) OR 题名或关键词=推拿) OR 题名或关键词=功法) OR 题名或关键词=易筋经) OR 题名或关键词=八段锦) OR 题名或关键词=太极) AND ((题名或关键词=认知障碍 OR 题名或关键词=认知衰退) OR 题名或关键词=轻度认知障碍))](https://qikan.cqvip.com/Qikan/search/index?LngMySearHistoryIdGuid=93bb252e-662c-41f3-b5c6-adf9a20256d4&from=Qikan_Article_History) |

## **Supplementary Table 2** Risk of bias and quality of evidence assessment.

| **Assessment Tool** | **Assessment Domains** | **Assessment Levels for Each Domain** | **Overall Rating** |
| --- | --- | --- | --- |
| the Cochrane Risk of Bias tool version 2.0 (RoB 2.0) | Randomization process、Deviations from intended interventions、Missing outcome data、Measurement of the outcome、Selection of the reported result | Low risk of bias、Some concerns、High risk of bias | Low risk of bias、Some concerns、High risk of bias |
| Confidence in Network Meta-Analysis(CINeMA) | Within-study bias、Reporting bias、Indirectness、Imprecision、Heterogeneity、Incoherence | No concerns、Low risk、Some concerns、Major concerns | High、Moderate、Low、Very low |

## **Supplementary Table 3** Definition of intervention measures and control group.

| Intervening measure | Definition |
| --- | --- |
| Manual acupuncture (MA) | Acupuncture involves manipulating specially crafted fine needles to penetrate specific acupoints on the body, stimulating these points through techniques such as lifting and thrusting, or twisting and rotating. |
| Electroacupuncture (EA) | After inserting the fine needle into an acupoint and obtaining the Qi sensation, an electroacupuncture device is connected to the needle handle. This device delivers pulsed electrical currents through the needle to target specific areas of the body. |
| Electroacupuncture (MOX) | After igniting moxa sticks or cones made from moxa wool, the resulting warmth is applied to specific acupoints or areas on the body's surface. Through its thermal effect and the pharmacological properties of mugwort, it regulates the body's meridians, qi and blood circulation, and organ functions. |
| Tuina (TN) | Tuina is a technique guided by Traditional Chinese Medicine theory, applying manual manipulation to specific areas or acupoints on the body's surface to regulate bodily functions. |
| Mind-Body exercise (MBE) | It is a form of exercise that organically integrates physical exercise with mental regulation, such as Baduanjin and Tai Chi. |
| Transcutaneous electrical acupoint stimulation (TEAS) | By placing electrode pads on specific acupoints on the skin surface and applying low-frequency pulsed currents, it simulates the stimulation effect of traditional acupuncture needle insertion to regulate bodily functions. |
| Acupoint catgut embedding (ACE) | Implanting absorbable medical sutures (such as catgut) into specific acupoints to provide sustained, gentle stimulation to the points. |
| Warm-needling acupuncture (WA) | After obtaining the qi sensation through acupuncture, secure moxa wool or moxa stick segments to the needle handle and ignite them. The heat generated by moxibustion is conducted through the needle shaft to the deeper layers of the acupoint, thereby achieving the dual effects of both acupuncture and moxibustion. |
| Auricular therapy (AT) | This is a method for both diagnosis and treatment, achieved by stimulating specific areas of the auricle (known as ear acupoints) through techniques such as pressing with Wangbuliuxing seeds, massage, or acupuncture. |
| Control group (CON) | The control group receives sham acupuncture, conventional drug treatment, maintenance of the original lifestyle, health education, or non-pharmacological traditional Chinese medicine interventions different from those of the treatment group. |

## **Supplementary Table 4** Characteristics of included studies.

| **Author** | **Year** | **Countey** | **Mean age** | **Sample size** | **Treatment** | **Control** | **Study duration (weeks)** | **Outcomes** |
| --- | --- | --- | --- | --- | --- | --- | --- | --- |
| Gong J | 2025 | China | T: 70.78 ± 4.15; C: 73.41 ± 4.95 | T: 27; C: 29 | MA | CON | 12 | MoCA |
| Jiang P | 2025 | China | T: 69 ± 5; C: 71 ± 6 | T: 29; C: 31 | MA | CON | 12 | MMSE; MoCA |
| Zhang Z | 2024 | China | T: 63.36 ± 2.89; C: 64.12 ± 2.56 | T: 48; C: 48 | MA | CON | 8 | MMSE; MoCA |
| Xu H | 2024 | China | T: 57.04 ± 3.18; C: 66.24 ± 3.55 | T: 40; C: 40 | MA | CON | 8 | MoCA |
| Qiao Y | 2024 | China | T: 67.44 ± 6.58; C: 67.81 ± 6.43 | T: 20; C: 20 | MA | CON | 4 | MMSE; MoCA |
| Chen Y | 2024 | China | T: 63.25 ± 2.75; C: 64.12 ± 2.56 | T: 32; C: 32 | MA | CON | 8 | MMSE; MoCA |
| Nie D | 2023 | China | T: 60.0 (52.3, 63.8); C: 58 (54, 69) | T: 40; C: 35 | MA | CON | 8 | MMSE; MoCA |
| Shi Z | 2023 | China | T: 62.44 ± 3.05; C: 63.12 ± 2.32 | T: 45; C: 45 | MA | CON | 12 | MMSE; MoCA |
| Lu C | 2023 | China | T: 60.5 (53.25, 65); C: 60.5 (55, 69) | T: 28; C: 28 | MA | CON | 8 | MMSE; MoCA |
| Li Y | 2023 | China | T: 60.5 ± 7.4; C: 62.8 ± 7.7 | T: 30; C: 30 | MA | CON | 4 | MMSE; MoCA |
| Liang J | 2022 | China | T: 64.03 ± 3.15; C: 63.15 ± 2.78 | T: 30; C: 27 | MA | CON | 12 | MoCA |
| Yuan X | 2021 | China | T: 64.05 ± 1.27; C: 63.15 ± 1.29 | T: 20; C: 20 | MA | CON | 8 | MMSE; MoCA |
| Zhang J | 2021 | China | T: 64.59 ± 7.74; C: 61.83 ± 7.37 | T: 17; C: 18 | MA | CON | 12 | MMSE; MoCA |
| Chen Y | 2019 | China | T: 71 ± 5; C: 71 ± 5 | T: 32; C: 32 | MA | CON | 8 | MMSE; MoCA |
| Chen J | 2019 | China | T: 67.77 ± 5.96; C: 70.23 ± 6.92 | T: 30; C: 30 | MA | CON | 4 | MMSE; MoCA |
| Zhou C | 2018 | China | T: 70.82 ± 8.39; C: 68.36 ± 6.67 | T: 74; C: 74 | MA | CON | 12 | MMSE; MoCA |
| Du L | 2015 | China | T: 69.03 ± 5.47; C: 68.25 ± 5.80 | T: 20; C: 20 | MA | CON | 8 | MMSE; MoCA |
| Sun Y | 2009 | China | T: 67.2 ± 4.3; C: 67.2 ± 4.3 | T: 29; C: 30 | MA | CON | 4 | MMSE |
| Feng C | 2009 | China | T: 60.2 ± 3.1; C: 62.5 ± 3.6 | T: 50; C: 49 | MA | CON | 12 | MMSE |
| Peng Z | 2016 | China | T: 65.7 ± 4.2; C: 64.9 ± 5.2 | T: 31; C: 32 | MA | CON | 8 | MMSE |
| Zhang J | 2025 | China | T: 59.03 ± 6.84; C: 61.71 ± 7.5 | T: 29; C: 24 | MA | CON | 8 | MMSE |
| Tan T | 2017 | China | T: 65.88 ± 4.66; C: 64.56 ± 5.25 | T: 16; C: 16 | MA | CON | 4 | MMSE; MoCA |
| Wang D | 2023 | China | T: 62.7 ± 7.8; C: 63.3 ± 7.4 | T: 24; C: 23 | EA | CON | 8 | MoCA |
| Yu P | 2021 | China | T: 65.48 ± 7.47; C: 63.32 ± 8.61 | T: 25; C: 25 | EA | CON | 8 | MMSE; MoCA |
| Xu J | 2017 | China | T: 62.12 ± 8.01; C: 61.20 ± 7.63 | T: 30; C: 30 | EA | CON | 8 | MMSE; MoCA |
| Liu J | 2016 | China | T: 73 ± 7.672; C: 77.22 ± 5.652 | T: 15; C: 17 | EA | CON | 4 | MMSE |
| Li X | 2013 | China | T: 62.8 ± 5.9; C: 61.9 ± 6.8 | T: 39; C: 39 | EA | CON | 8 | MMSE; MoCA |
| Luo Z | 2013 | China | —— | T: 40; C: 40 | EA | CON | 8 | MMSE |
| Zhao Y | 2012 | China | T: 72.68 ± 8.26; C: 72.96 ± 4.95 | T: 25; C: 26 | EA | CON | 8 | MMSE |
| Chen Z | 2011 | China | T: 71.28 ± 7.834; C: 72.32 ± 9.371 | T: 80; C: 75 | EA | CON | 8 | MMSE |
| Liu X | 2010 | China | T: 66.00 ± 6.84; C: 69.32 ± 6.86 | T: 17; C: 19 | EA | CON | 4 | MMSE; MoCA |
| Jin X | 2010 | China | —— | T: 14; C: 16 | EA | CON | 6.43 | MoCA |
| Yu X | 2010 | China | T: 68.309 ± 7.333; C: 70.170 ± 4.475 | T: 55; C: 53 | EA | CON | 8 | MMSE |
| Kim J H | 2020 | Korea | T: 69.94 ± 5.94; C: 74.25 ± 5.39 | T: 16; C: 16 | EA | CON | 8 | MoCA |
| Liang Y | 2024 | China | T: 61.55 ± 5.82; C: 60.24 ± 5.63 | T: 40; C: 40 | MOX | CON | 4 | MoCA |
| Shi J | 2024 | China | T: 61.3 ± 5.2; C: 59.1 ± 4.1 | T: 18; C: 16 | MOX | CON | 8 | MMSE; MoCA |
| Wang H | 2022 | China | T: 65.1 ± 8.7; C: 63.4 ± 9.3 | T: 52; C: 51 | MOX | CON | 8 | MMSE; MoCA |
| Wang H | 2021 | China | T: 72.3 ± 8.6; C: 70.3 ± 9.4 | T: 49; C: 46 | MOX | CON | 8 | MMSE; MoCA |
| Liu Q | 2020 | China | T: 62 ± 5; C: 60 ± 4 | T: 28; C: 26 | MOX | CON | 8 | MMSE; MoCA |
| Li S | 2020 | China | T: 65.10 ± 8.76; C: 61.14 ± 9.05 | T: 31; C: 29 | MOX | CON | 8 | MMSE; MoCA |
| Su J | 2019 | China | T: 63.85 ± 6.32; C: 67.05 ± 6.10 | T: 26; C: 21 | MOX | CON | 8 | MMSE; MoCA |
| Zhao L | 2019 | China | T: 64.13 ± 6.02; C: 66.75 ± 6.41 | T: 31; C: 20 | MOX | CON | 8 | MMSE; MoCA |
| Jia X | 2017 | China | T: 61.69 ± 4.74; C: 59.63 ± 3.90 | T: 30; C: 30 | MOX | CON | 8 | MoCA |
| Liu C | 2017 | China | T: 72.33 ± 8.363; C: 70.48 ± 9.387 | T: 63; C: 60 | MOX | CON | 8 | MMSE; MoCA |
| Li X | 2023 | China | —— | T: 16; C: 16 | MOX | CON | 8 | MMSE; MoCA |
| Liu C | 2022 | China | T: 64.07 ± 6.07; C: 64.25 ± 7.50 | T: 27; C: 20 | MOX | CON | 8 | MMSE; MoCA |
| Wang J | 2025 | China | T: 68.40 ± 3.19; C: 67.57 ± 3.43 | T: 30; C: 30 | TN | CON | 24 | MMSE; MoCA |
| Peng Y | 2020 | China | T: 71.16 ± 4.40; C: 70.76 ± 3.64 | T: 26; C: 25 | TN | CON | 12 | MoCA |
| Sun J | 2015 | China | T: 70.8 ± 6.5; C: 70.3 ± 3.9 | T: 38; C: 38 | TN | CON | 24 | MMSE; MoCA |
| Feng X | 2015 | China | T+C: 67.83 ± 4.76 | T: 50; C: 50 | TN | CON | 12 | MMSE |
| Zheng Y | 2025 | China | T: 59.00 ± 5.52; C: 59.20 ± 5.24 | T: 20; C: 20 | MBE | CON | 12 | MMSE |
| Xu W | 2023 | China | T: 67.5 ± 7.3; C: 68.6 ± 7.5 | T: 31; C: 32 | MBE | CON | 12 | MoCA |
| Sun Z | 2021 | China | —— | T: 29; C: 28 | MBE | CON | 24 | MoCA |
| Liu H | 2020 | China | —— | T: 28; C: 30 | MBE | CON | 24 | MoCA |
| Liu T | 2018 | China | T: 71.23 ± 5.53; C: 71.60 ± 5.29 | T: 29; C: 28 | MBE | CON | 24 | MoCA |
| Li M | 2017 | China | T: 66.16 ± 4.16; C: 65.41 ± 4.90 | T: 36; C: 32 | MBE | CON | 24 | MoCA |
| Li S | 2016 | China | T: 66.59 ± 4.02; C: 65.93 ± 5.13 | T: 28; C: 29 | MBE | CON | 24 | MoCA |
| Zhou C | 2025 | China | T: 66.05 ± 6.64; C: 66.90 ± 4.94 | T: 22; C: 22 | MBE | CON | 12 | MMSE; MoCA |
| Lin L | 2025 | China | T: 86.17 ± 6.022; C: 81.96 ± 6.12 | T: 18; C: 18 | MBE | CON | 12 | MoCA |
| Lin M | 2024 | China | T: 67.38 ± 3.91; C: 68.38 ± 4.13 | T: 48; C: 48 | MBE | CON | 12 | MoCA |
| Li F | 2022 | United States | T: 74.5  ±  5.6; C: 74.9  ±  6.3 | T: 22; C: 24 | MBE | CON | 16 | MoCA |
| Liu C | 2022 | China | T: 73.2 ±  6.3; C: 73.4 ±  6.5 | T: 17; C: 17 | MBE | CON | 12 | MoCA |
| Yu A P | 2022 | China | T: 67.3 ± 4.2; C: 67.6 ± 8.1 | T: 10; C: 12 | MBE | CON | 24 | MoCA |
| Liu J | 2021 | China | T: 66.17 ± 4.17; C: 65.97 ± 5.66 | T: 20; C: 20 | MBE | CON | 24 | MoCA |
| Zheng G | 2021 | China | T: 65.79 ± 4.35; C: 65.86 ± 5.28 | T: 20; C: 20 | MBE | CON | 24 | MoCA |
| Xu W | 2024 | China | T: 77 (73.25, 79); C: 76.5 (73, 79) | T: 34; C: 32 | TEAS | CON | 12 | MoCA |
| Hu Y | 2023 | China | T: 56.87 ± 10.28; C: 55.41 ± 12.56 | T: 78; C: 78 | TEAS | CON | 12 | MoCA |
| Xu W | 2024 | China | T: 77 (71.75, 78); C: 77 (73, 79) | T: 32; C: 29 | TEAS | CON | 12 | MoCA |
| Shang S | 2018 | China | —— | T: 40; C: 40 | ACE | CON | 12 | MMSE |
| Jin X Y | 2010 | China | T: 77.28 ± 5.59; C: 76.96 ± 6.56 | T: 25; C: 25 | ACE | CON | 24 | MMSE |
| He X | 2018 | China | T: 64 ± 7; C: 66 ± 6 | T: 30; C: 30 | WA | CON | 12 | MMSE; MoCA |
| Zhu C | 2015 | China | T: 62.13 ± 7.99; C: 60.90 ± 7.52 | T: 30; C: 30 | WA | CON | 8 | MMSE; MoCA |
| Xu G | 2020 | China | T: 74.6 ± 6.1; C: 74.2 ± 6.3 | T: 30; C: 30 | AT | CON | 12 | MMSE |
| Sun X | 2019 | China | T: 67.63 ± 6.06; C: 67.63 ± 6.06 | T: 80; C: 80 | AT | CON | 48 | MMSE; MoCA |
| Chen J | 2019 | China | T: 73.9 ± 5.98; C: 75.30 ± 5.44 | T: 31; C: 30 | AT | CON | 20 | MMSE; MoCA |
| Wang Y | 2021 | China | T: 60.52 ± 6.05; C: 59.35 ± 6.17 | T: 32; C: 32 | ACE | MA | 8 | MMSE; MoCA |

MA, manual acupuncture; EA, electroacupuncture; MOX, moxibustion; TN, tuina; MBE, mind-body exercise; TEAS, transcutaneous electrical acupoint stimulation; ACE, acupoint catgut embedding; WA, warm-needling acupuncture; AT, auricular therapy; CON, control group. MMSE: mini-mental state examination; MoCA: montreal cognitive assessment.

## **Supplementary Table 5** CINeMA evidence quality assessment.

## **Supplementary Table 5.1** Reasons for downgrading MMSE.

| Comparison | Number of studies | Within-study bias | Reporting bias | Indirectness | Imprecision | Heterogeneity | Incoherence | Confidence rating |
| --- | --- | --- | --- | --- | --- | --- | --- | --- |
| ACE:CON | 2 | Some concerns | Low risk | No concerns | No concerns | No concerns | No concerns | Moderate |
| ACE:MA | 1 | No concerns | Low risk | No concerns | No concerns | No concerns | No concerns | High |
| AT:CON | 3 | No concerns | Low risk | No concerns | No concerns | Some concerns | No concerns | Moderate |
| CON:EA | 9 | Some concerns | Low risk | No concerns | No concerns | Some concerns | No concerns | Low |
| CON:MA | 19 | No concerns | Low risk | No concerns | No concerns | No concerns | No concerns | High |
| CON:MBE | 2 | No concerns | Low risk | No concerns | No concerns | No concerns | No concerns | High |
| CON:MOX | 10 | No concerns | Low risk | No concerns | No concerns | Some concerns | No concerns | Moderate |
| CON:TN | 3 | Some concerns | Low risk | No concerns | No concerns | No concerns | No concerns | Moderate |
| CON:WA | 2 | Some concerns | Low risk | No concerns | No concerns | Some concerns | No concerns | Low |
| ACE:AT | 0 | Some concerns | Low risk | No concerns | No concerns | Some concerns | No concerns | Low |
| ACE:EA | 0 | Some concerns | Low risk | No concerns | No concerns | Some concerns | No concerns | Low |
| ACE:MBE | 0 | No concerns | Low risk | No concerns | No concerns | Some concerns | No concerns | Moderate |
| ACE:MOX | 0 | Some concerns | Low risk | No concerns | No concerns | Some concerns | No concerns | Low |
| ACE:TN | 0 | Some concerns | Low risk | No concerns | No concerns | No concerns | No concerns | Moderate |
| ACE:WA | 0 | Some concerns | Low risk | No concerns | Some concerns | No concerns | No concerns | Low |
| AT:EA | 0 | No concerns | Low risk | No concerns | No concerns | No concerns | No concerns | High |
| AT:MA | 0 | No concerns | Low risk | No concerns | No concerns | Some concerns | No concerns | Moderate |
| AT:MBE | 0 | No concerns | Low risk | No concerns | Some concerns | No concerns | No concerns | Moderate |
| AT:MOX | 0 | No concerns | Low risk | No concerns | No concerns | No concerns | No concerns | High |
| AT:TN | 0 | Some concerns | Low risk | No concerns | No concerns | Some concerns | No concerns | Low |
| AT:WA | 0 | Some concerns | Low risk | No concerns | No concerns | No concerns | No concerns | Moderate |
| EA:MA | 0 | No concerns | Low risk | No concerns | No concerns | Some concerns | No concerns | Moderate |
| EA:MBE | 0 | No concerns | Low risk | No concerns | Some concerns | No concerns | No concerns | Moderate |
| EA:MOX | 0 | No concerns | Low risk | No concerns | No concerns | No concerns | No concerns | High |
| EA:TN | 0 | Some concerns | Low risk | No concerns | No concerns | Some concerns | No concerns | Low |
| EA:WA | 0 | Some concerns | Low risk | No concerns | No concerns | No concerns | No concerns | Moderate |
| MA:MBE | 0 | No concerns | Low risk | No concerns | No concerns | Some concerns | No concerns | Moderate |
| MA:MOX | 0 | No concerns | Low risk | No concerns | No concerns | Some concerns | No concerns | Moderate |
| MA:TN | 0 | Some concerns | Low risk | No concerns | No concerns | No concerns | No concerns | Moderate |
| MA:WA | 0 | Some concerns | Low risk | No concerns | No concerns | Some concerns | No concerns | Low |
| MBE:MOX | 0 | No concerns | Low risk | No concerns | Some concerns | No concerns | No concerns | Moderate |
| MBE:TN | 0 | Some concerns | Low risk | No concerns | No concerns | Some concerns | No concerns | Low |
| MBE:WA | 0 | Some concerns | Low risk | No concerns | Some concerns | No concerns | No concerns | Low |
| MOX:TN | 0 | Some concerns | Low risk | No concerns | No concerns | Some concerns | No concerns | Low |
| MOX:WA | 0 | Some concerns | Low risk | No concerns | No concerns | No concerns | No concerns | Moderate |
| TN:WA | 0 | Some concerns | Low risk | No concerns | Some concerns | No concerns | No concerns | Low |

## MA, manual acupuncture; EA, electroacupuncture; MOX, moxibustion; TN, tuina; MBE, mind-body exercise; TEAS, transcutaneous electrical acupoint stimulation; ACE, acupoint catgut embedding; WA, warm-needling acupuncture; AT, auricular therapy; CON, control group.

## **Supplementary Table 5.2** Reasons for downgrading MoCA.

| Comparison | Number of studies | Within-study bias | Reporting bias | Indirectness | Imprecision | Heterogeneity | Incoherence | Confidence rating |
| --- | --- | --- | --- | --- | --- | --- | --- | --- |
| ACE:MA | 1 | No concerns | Low risk | No concerns | Some concerns | No concerns | Major concerns | Very low |
| AT:CON | 2 | No concerns | Low risk | No concerns | Some concerns | No concerns | Major concerns | Very low |
| CON:EA | 7 | Some concerns | Low risk | No concerns | No concerns | Some concerns | Major concerns | Very low |
| CON:MA | 18 | No concerns | Low risk | No concerns | No concerns | No concerns | Major concerns | Low |
| CON:MBE | 14 | No concerns | Low risk | No concerns | No concerns | Some concerns | Major concerns | Very low |
| CON:MOX | 12 | No concerns | Low risk | No concerns | No concerns | Some concerns | Major concerns | Very low |
| CON:TEAS | 3 | Some concerns | Low risk | No concerns | No concerns | No concerns | Major concerns | Very low |
| CON:TN | 3 | No concerns | Low risk | No concerns | No concerns | No concerns | Major concerns | Low |
| CON:WA | 2 | Some concerns | Low risk | No concerns | Some concerns | No concerns | Major concerns | Very low |
| ACE:AT | 0 | No concerns | Low risk | No concerns | Some concerns | No concerns | Major concerns | Very low |
| ACE:CON | 0 | No concerns | Low risk | No concerns | No concerns | No concerns | Major concerns | Low |
| ACE:EA | 0 | No concerns | Low risk | No concerns | No concerns | Some concerns | Major concerns | Very low |
| ACE:MBE | 0 | No concerns | Low risk | No concerns | Some concerns | No concerns | Major concerns | Very low |
| ACE:MOX | 0 | No concerns | Low risk | No concerns | Some concerns | No concerns | Major concerns | Very low |
| ACE:TEAS | 0 | No concerns | Low risk | No concerns | Major concerns | No concerns | Major concerns | Very low |
| ACE:TN | 0 | No concerns | Low risk | No concerns | Major concerns | No concerns | Major concerns | Very low |
| ACE:WA | 0 | No concerns | Low risk | No concerns | Some concerns | No concerns | Major concerns | Very low |
| AT:EA | 0 | Some concerns | Low risk | No concerns | Some concerns | Some concerns | Major concerns | Very low |
| AT:MA | 0 | No concerns | Low risk | No concerns | Some concerns | No concerns | Major concerns | Very low |
| AT:MBE | 0 | No concerns | Low risk | No concerns | Some concerns | No concerns | Major concerns | Very low |
| AT:MOX | 0 | No concerns | Low risk | No concerns | Some concerns | Some concerns | Major concerns | Very low |
| AT:TEAS | 0 | Some concerns | Low risk | No concerns | No concerns | Some concerns | Major concerns | Very low |
| AT:TN | 0 | No concerns | Low risk | No concerns | Some concerns | No concerns | Major concerns | Very low |
| AT:WA | 0 | Some concerns | Low risk | No concerns | Some concerns | Some concerns | Major concerns | Very low |
| EA:MA | 0 | No concerns | Low risk | No concerns | No concerns | Some concerns | Major concerns | Very low |
| EA:MBE | 0 | No concerns | Low risk | No concerns | No concerns | Some concerns | Major concerns | Very low |
| EA:MOX | 0 | Some concerns | Low risk | No concerns | Some concerns | No concerns | Major concerns | Very low |
| EA:TEAS | 0 | Some concerns | Low risk | No concerns | No concerns | Some concerns | Major concerns | Very low |
| EA:TN | 0 | Some concerns | Low risk | No concerns | No concerns | Some concerns | Major concerns | Very low |
| EA:WA | 0 | Some concerns | Low risk | No concerns | No concerns | Major concerns | Major concerns | Very low |
| MA:MBE | 0 | No concerns | Low risk | No concerns | No concerns | Major concerns | Major concerns | Very low |
| MA:MOX | 0 | No concerns | Low risk | No concerns | No concerns | Some concerns | Major concerns | Very low |
| MA:TEAS | 0 | No concerns | Low risk | No concerns | Some concerns | No concerns | Major concerns | Very low |
| MA:TN | 0 | No concerns | Low risk | No concerns | Some concerns | No concerns | Major concerns | Very low |
| MA:WA | 0 | Some concerns | Low risk | No concerns | Some concerns | No concerns | Major concerns | Very low |
| MBE:MOX | 0 | No concerns | Low risk | No concerns | No concerns | Some concerns | Major concerns | Very low |
| MBE:TEAS | 0 | No concerns | Low risk | No concerns | Some concerns | No concerns | Major concerns | Very low |
| MBE:TN | 0 | No concerns | Low risk | No concerns | Some concerns | No concerns | Major concerns | Very low |
| MBE:WA | 0 | Some concerns | Low risk | No concerns | Some concerns | No concerns | Major concerns | Very low |
| MOX:TEAS | 0 | Some concerns | Low risk | No concerns | No concerns | Some concerns | Major concerns | Very low |
| MOX:TN | 0 | No concerns | Low risk | No concerns | Some concerns | No concerns | Major concerns | Very low |
| MOX:WA | 0 | Some concerns | Low risk | No concerns | Some concerns | No concerns | Major concerns | Very low |
| TEAS:TN | 0 | Some concerns | Low risk | No concerns | Some concerns | Some concerns | Major concerns | Very low |
| TEAS:WA | 0 | Some concerns | Low risk | No concerns | No concerns | Some concerns | Major concerns | Very low |
| TN:WA | 0 | Some concerns | Low risk | No concerns | No concerns | Some concerns | Major concerns | Very low |

MA, manual acupuncture; EA, electroacupuncture; MOX, moxibustion; TN, tuina; MBE, mind-body exercise; TEAS, transcutaneous electrical acupoint stimulation; ACE, acupoint catgut embedding; WA, warm-needling acupuncture; AT, auricular therapy; CON, control group.

## **Supplementary Table 6** League table for short-term intervention.

| MA | EA | MOX | TN | MBE | TEAS | ACE | WA | AT | CON |
| --- | --- | --- | --- | --- | --- | --- | --- | --- | --- |
| MA | 0.47 (-0.24, 1.19) | 0.59 (-0.1, 1.29) | 0.33 (-3.17, 3.58) | -0.21 (-20.78, 27.36) | — | -0.38 (-1.91, 1.17) | 0.83 (-0.76, 2.41) | -0.46 (-3.05, 2.03) | **1.66 (1.2, 2.12)** |
| **1.65 (0.45, 2.84)** | EA | 0.12 (-0.63, 0.87) | -0.13 (-3.65, 3.13) | -0.69 (-21.36, 26.83) | — | -0.85 (-2.51, 0.83) | 0.36 (-1.27, 1.97) | -0.92 (-3.54, 1.58) | **1.19 (0.64, 1.73)** |
| 0.63 (-0.39, 1.63) | -1.02 (-2.22, 0.19) | MOX | -0.25 (-3.75, 3.02) | -0.81 (-21.46, 26.72) | — | -0.97 (-2.63, 0.7) | 0.24 (-1.37, 1.84) | -1.04 (-3.63, 1.45) | **1.07 (0.55, 1.58)** |
| 3.31 (-1.53, 8) | 1.65 (-3.21, 6.33) | 2.67 (-2.13, 7.32) | TN | -0.62 (-21.75, 27.13) | — | -0.72 (-4.34, 3.12) |  | -0.78 (-4.93, 3.38) | 1.32 (-1.9, 4.81) |
| 0.47 (-1.88, 2.92) | -1.17 (-3.63, 1.38) | -0.16 (-2.54, 2.31) | -2.81 (-7.98, 2.46) | MBE | — | -0.15 (-27.61, 20.53) | 1.04 (-26.4, 21.73) | -0.28 (-28.09, 20.34) | 1.87 (-25.67, 22.49) |
| -0.35 (-25.93, 44.75) | -1.98 (-27.81, 42.99) | -0.97 (-26.71, 44.05) | -3.47 (-29.8, 41.86) | -0.73 (-26.63, 44.41) | TEAS | — | — | — | — |
| -1.06 (-3.4, 1.28) | -2.71 (-5.34, -0.09) | -1.68 (-4.24, 0.85) | -4.37 (-9.56, 1) | -1.53 (-4.91, 1.74) | -0.75 (-45.79, 24.87) | ACE | 1.21 (-0.98, 3.41) | -0.07 (-3.1, 2.82) | **2.03 (0.45, 3.62)** |
| 1.97 (-0.31, 4.26) | 0.32 (-2.05, 2.71) | 1.34 (-0.94, 3.64) | -1.33 (-6.42, 3.83) | 1.49 (-1.69, 4.61) | 2.29 (-42.9, 28.3) | 3.03 (-0.26, 6.31) | WA | -1.29 (-4.23, 1.56) | 0.82 (-0.69, 2.35) |
| 0.08 (-4.73, 4.75) | -1.57 (-6.44, 3.12) | -0.54 (-5.37, 4.1) | -3.24 (-9.86, 3.55) | -0.41 (-5.72, 4.75) | 0.36 (-44.16, 25.01) | 1.14 (-4.16, 6.39) | -1.9 (-7.1, 3.22) | AT | 2.11 (-0.32, 4.66) |
| **2.43 (1.73, 3.13)** | 0.79 (-0.18, 1.75) | **1.8 (1.08, 2.53)** | -0.87 (-5.46, 3.88) | 1.96 (-0.41, 4.24) | 2.77 (-42.23, 28.5) | **3.49 (1.05, 5.94)** | 0.46 (-1.72, 2.64) | 2.35 (-2.25, 7.14) | CON |

The upper-right triangle corresponds to MMSE outcomes (row vs. column), while the lower-left triangle corresponds to MoCA outcomes (column vs. row). Statistically significant results are highlighted in bold. MA, manual acupuncture; EA, electroacupuncture; MOX, moxibustion; TN, tuina; MBE, mind-body exercise; TEAS, transcutaneous electrical acupoint stimulation; ACE, acupoint catgut embedding; WA, warm-needling acupuncture; AT, auricular therapy; CON, control group.

## **Supplementary Table 7** League table for medium-term intervention.

| MA | EA | MOX | TN | MBE | TEAS | ACE | WA | AT | CON |
| --- | --- | --- | --- | --- | --- | --- | --- | --- | --- |
| MA | 0.38 (-14.4, 10.77) | 0.31 (-13.52, 14.37) | 0.33 (-1.46, 2.05) | -0.17 (-1.76, 1.35) | — | 0.22 (-1.03, 1.43) | 0.63 (-1.01, 2.25) | 0.62 (-0.7, 1.89) | **2.04 (1.29, 2.76)** |
| 2.5 (-6.36, 18.85) | EA | 0.21 (-16.74, 18.98) | -0.09 (-10.27, 14.82) | -0.56 (-10.95, 14.39) | — | -0.18 (-10.56, 14.65) | 0.25 (-10.22, 15.18) | 0.19 (-10.09, 15.09) | 1.65 (-8.68, 16.46) |
| -0.27 (-13.63, 9.51) | -2.98 (-24.68, 9.46) | MOX | 0.08 (-14.13, 13.89) | -0.45 (-14.58, 13.45) | — | -0.06 (-14.17, 13.8) | 0.33 (-13.73, 14.28) | 0.35 (-13.77, 14.17) | 1.73 (-12.37, 15.62) |
| 0.2 (-2.25, 2.58) | -2.34 (-18.79, 6.45) | 0.46 (-8.96, 13.65) | TN | -0.49 (-2.62, 1.6) | — | -0.11 (-1.99, 1.8) | 0.3 (-1.87, 2.5) | 0.29 (-1.62, 2.19) | **1.71 (0.14, 3.35)** |
| -0.34 (-1.81, 1.11) | -2.77 (-18.99, 6.02) | -0.05 (-9.74, 13.24) | -0.54 (-2.99, 1.94) | MBE | — | 0.38 (-1.31, 2.12) | 0.79 (-1.18, 2.84) | 0.78 (-0.94, 2.55) | **2.21 (0.85, 3.62)** |
| -1.63 (-3.35, 0.1) | -4.08 (-20.28, 4.69) | -1.34 (-11.09, 11.98) | -1.82 (-4.43, 0.84) | -1.28 (-3.03, 0.5) | TEAS | — | — | — | — |
| -1.89 (-21.25, 15.2) | -4.8 (-26.38, 12.69) | -1.43 (-24.51, 21.6) | -2.05 (-21.85, 14.87) | -1.55 (-20.82, 15.59) | -0.25 (-19.63, 16.8) | ACE | 0.41 (-1.35, 2.2) | 0.4 (-1.06, 1.84) | **1.82 (0.83, 2.83)** |
| 0.51 (-1.89, 2.89) | -1.98 (-18.47, 7.15) | 0.81 (-8.88, 14.11) | 0.3 (-2.78, 3.52) | 0.86 (-1.59, 3.28) | 2.13 (-0.48, 4.74) | 2.43 (-14.65, 21.73) | WA | -0.01 (-1.86, 1.78) | 1.41 (-0.05, 2.87) |
| 0.17 (-2.18, 2.54) | -2.36 (-18.5, 6.58) | 0.43 (-9.31, 13.91) | -0.03 (-3.1, 3.14) | 0.52 (-1.88, 2.92) | 1.8 (-0.76, 4.38) | 2.08 (-14.88, 21.43) | -0.34 (-3.4, 2.73) | AT | **1.43 (0.39, 2.49)** |
| **1.69 (0.71, 2.67)** | -0.74 (-16.87, 7.9) | 1.94 (-7.68, 15.19) | 1.49 (-0.7, 3.76) | **2.04 (0.97, 3.09)** | **3.32 (1.9, 4.72)** | 3.57 (-13.47, 22.9) | 1.18 (-1.01, 3.38) | 1.52 (-0.64, 3.67) | CON |

The upper-right triangle corresponds to MMSE outcomes (row vs. column), while the lower-left triangle corresponds to MoCA outcomes (column vs. row). Statistically significant results are highlighted in bold. MA, manual acupuncture; EA, electroacupuncture; MOX, moxibustion; TN, tuina; MBE, mind-body exercise; TEAS, transcutaneous electrical acupoint stimulation; ACE, acupoint catgut embedding; WA, warm-needling acupuncture; AT, auricular therapy; CON, control group.

## **Supplementary Table 8** League table for long-term intervention.

| MA | EA | MOX | TN | MBE | TEAS | ACE | WA | AT | CON |
| --- | --- | --- | --- | --- | --- | --- | --- | --- | --- |
| MA | 0.27 (-29.26, 21.04) | 0.04 (-27.65, 28.1) | 0.33 (-1.62, 2.23) | -0.15 (-27.48, 20.34) | — | 0.82 (-1.43, 2.96) | 0.42 (-3.12, 3.97) | 1.69 (-0.46, 3.8) | **2.44 (0.86, 3.89)** |
| 3.35 (-14.32, 36.1) | EA | 0.31 (-33.58, 37.87) | 0.05 (-20.83, 29.81) | -0.5 (-30.71, 32.84) | — | 0.51 (-20.25, 30.44) | 0.15 (-20.95, 30.17) | 1.42 (-19.3, 31) | 2.12 (-18.52, 31.7) |
| -1.17 (-28, 18.38) | -4.95 (-48.3, 19.83) | MOX | 0.31 (-27.91, 28.05) | -0.89 (-30.1, 28.72) | — | 0.82 (-27.42, 28.51) | 0.39 (-27.67, 28.42) | 1.67 (-26.61, 29.44) | 2.38 (-25.84, 30.14) |
| **-2.91 (-5.63, -0.14)** | -6.13 (-38.3, 11.26) | -1.75 (-21.22, 24.9) | TN | -0.44 (-27.81, 20.24) | — | 0.49 (-1.48, 2.45) | 0.11 (-3.31, 3.46) | 1.36 (-0.52, 3.25) | **2.1 (0.94, 3.24)** |
| -1.17 (-3.44, 1.04) | -4.38 (-36.43, 12.76) | -0.06 (-19.2, 26.42) | 1.74 (-0.25, 3.66) | MBE | — | 0.95 (-19.67, 28.21) | 0.56 (-20.59, 27.82) | 1.84 (-18.78, 29.28) | 2.56 (-18.01, 29.85) |
| -2.88 (-48.2, 22.44) | -6.7 (-57.37, 18.94) | -1.6 (-49.62, 30.31) | -0.01 (-45.01, 25.91) | -1.75 (-46.7, 24.03) | TEAS | — | — | — | — |
| -2.7 (-41.29, 31.34) | -6.87 (-49.83, 27.67) | -1.18 (-47.27, 44.84) | 0.21 (-38.2, 34.3) | -1.51 (-40.13, 32.4) | 1.26 (-47.87, 48.79) | ACE | -0.38 (-3.95, 3.21) | 0.87 (-1.31, 3.08) | **1.62 (0.01, 3.2)** |
| -0.94 (-6.12, 4.11) | -4.29 (-37.42, 14.08) | 0.31 (-19.06, 26.86) | 1.98 (-3.08, 6.9) | 0.25 (-4.67, 4.96) | 2.09 (-24.57, 47.63) | 1.82 (-32.18, 40.19) | WA | 1.25 (-2.28, 4.77) | 1.99 (-1.22, 5.2) |
| 0.26 (-2.86, 3.41) | -2.92 (-35.55, 14.39) | 1.42 (-17.86, 27.98) | **3.18 (0.28, 6.02)** | 1.44 (-1.02, 3.95) | 3.18 (-22.13, 48) | 2.94 (-31.2, 41.61) | 1.2 (-3.99, 6.54) | AT | 0.74 (-0.78, 2.22) |
| 0.94 (-1.12, 3) | -2.23 (-34.42, 14.91) | 2.06 (-17.12, 28.53) | **3.86 (2.11, 5.56)** | **2.12 (1.2, 3.06)** | 3.85 (-21.96, 48.77) | 3.63 (-30.32, 42.27) | 1.87 (-2.75, 6.69) | 0.68 (-1.62, 2.98) | CON |

The upper-right triangle corresponds to MMSE outcomes (row vs. column), while the lower-left triangle corresponds to MoCA outcomes (column vs. row). Statistically significant results are highlighted in bold. MA, manual acupuncture; EA, electroacupuncture; MOX, moxibustion; TN, tuina; MBE, mind-body exercise; TEAS, transcutaneous electrical acupoint stimulation; ACE, acupoint catgut embedding; WA, warm-needling acupuncture; AT, auricular therapy; CON, control group.

**Supplementary Table 9** SUCRA rankings for MMSE scores by intervention duration.

| Rank | Short-term | SUCRA (%) | Medium-term | SUCRA (%) | Long-term | SUCRA (%) |
| --- | --- | --- | --- | --- | --- | --- |
| 1 | ACE | 74.08 | MBE | 70.56 | MA | 68.11 |
| 2 | AT | 70.67 | MA | 67.24 | TN | 61.82 |
| 3 | MA | 68.08 | ACE | 58.13 | MBE | 57.35 |
| 4 | MBE | 54.85 | TN | 54.29 | WA | 56.87 |
| 5 | TN | 50.65 | MOX | 52.1 | MOX | 53.28 |
| 6 | EA | 46.13 | EA | 51.24 | EA | 52.45 |
| 7 | MOX | 40.52 | AT | 43.39 | ACE | 50.76 |
| 8 | WA | 35.55 | WA | 44.3 | AT | 32.55 |
| 9 | CON | 9.46 | CON | 8.76 | CON | 16.8 |

MA, manual acupuncture; EA, electroacupuncture; MOX, moxibustion; TN, tuina; MBE, mind-body exercise; TEAS, transcutaneous electrical acupoint stimulation; ACE, acupoint catgut embedding; WA, warm-needling acupuncture; AT, auricular therapy; CON, control group.

**Supplementary Table 10** SUCRA rankings for MoCA scores by intervention duration.

| Rank | Short-term | SUCRA (%) | Medium-term | SUCRA (%) | Long-term | SUCRA (%) |
| --- | --- | --- | --- | --- | --- | --- |
| 1 | ACE | 0.8503 | TEAS | 0.8418 | TN | 0.7836 |
| 2 | MA | 0.7314 | ACE | 0.6473 | TEAS | 0.637 |
| 3 | AT | 0.6273 | MBE | 0.6095 | MBE | 0.5918 |
| 4 | MBE | 0.6029 | MOX | 0.5348 | ACE | 0.5735 |
| 5 | TEAS | 0.5846 | MA | 0.5224 | WA | 0.5297 |
| 6 | MOX | 0.5757 | AT | 0.4883 | MOX | 0.5211 |
| 7 | EA | 0.3528 | TN | 0.483 | MA | 0.414 |
| 8 | WA | 0.301 | WA | 0.4182 | AT | 0.3747 |
| 9 | TN | 0.2003 | EA | 0.2917 | EA | 0.3192 |
| 10 | CON | 0.1737 | CON | 0.163 | CON | 0.2554 |

MA, manual acupuncture; EA, electroacupuncture; MOX, moxibustion; TN, tuina; MBE, mind-body exercise; TEAS, transcutaneous electrical acupoint stimulation; ACE, acupoint catgut embedding; WA, warm-needling acupuncture; AT, auricular therapy; CON, control group.

**Supplementary Table 11** League table for aMCI subgroup.

| MA | EA | MOX | TN | MBE | TEAS | ACE | WA | AT | CON |
| --- | --- | --- | --- | --- | --- | --- | --- | --- | --- |
| MA | 1.11 (-0.37, 2.56) | -0.3 (-1.35, 0.73) | -0.19 (-23.83, 21.59) | -0.02 (-20.72, 43.25) | — | 0.34 (-23.61, 40) | 1.1 (-0.41, 2.59) | 1.41 (-16.03, 20.63) | **1.88 (1.33, 2.41)** |
| 1.99 (-0.45, 4.46) | EA | -1.41 (-3.04, 0.24) | -1.26 (-25.09, 20.39) | -1.13 (-21.98, 42.07) | — | -0.75 (-24.7, 38.91) | -0.01 (-1.93, 1.93) | 0.32 (-17.15, 19.57) | 0.77 (-0.6, 2.14) |
| -0.74 (-2.51, 1.05) | -1.99 (-4.46, 0.45) | MOX | 0.1 (-23.47, 21.9) | 0.29 (-20.4, 43.48) | — | 0.64 (-23.23, 40.18) | 1.4 (-0.25, 3.07) | 1.7 (-15.82, 20.98) | **2.18 (1.29, 3.08)** |
| -1.12 (-61.67, 49.22) | -3.21 (-63.89, 47.05) | -0.36 (-60.87, 50.02) | TN | 0.51 (-25.91, 44.86) | — | 0.65 (-30.32, 43.01) | 1.26 (-20.55, 25.07) | 2.21 (-28.98, 30.54) | 2.06 (-19.68, 25.72) |
| 0.5 (-1.46, 2.49) | -1.48 (-4.39, 1.37) | 1.25 (-1.1, 3.61) | 1.62 (-48.3, 62.35) | MBE | — | 0.3 (-42.95, 35.04) | 1.1 (-41.96, 21.75) | 1.16 (-40.43, 28.14) | 1.89 (-41.35, 22.59) |
| -0.84 (-2.85, 1.2) | -2.83 (-5.82, 0.11) | -0.09 (-2.49, 2.29) | 0.33 (-49.83, 60.86) | -1.34 (-3.91, 1.21) | TEAS | — | — | — | — |
| -0.38 (-49.13, 52.05) | -2.37 (-51.14, 50.27) | 0.34 (-48.17, 52.66) | 1.17 (-59.07, 66.49) | -0.91 (-49.31, 51.47) | 0.45 (-48.24, 52.79) | ACE | 0.78 (-38.78, 24.54) | 0.74 (-32.63, 33.12) | 1.54 (-38.1, 25.42) |
| 1.77 (-0.68, 4.24) | -0.21 (-3.51, 3) | 2.52 (-0.3, 5.28) | 2.97 (-47.5, 63) | 1.26 (-1.64, 4.17) | 2.61 (-0.31, 5.53) | 2.15 (-50.11, 50.82) | WA | 0.34 (-17.11, 19.53) | 0.77 (-0.62, 2.18) |
| 1.15 (-57.93, 38.49) | -0.85 (-60.2, 36.34) | 1.87 (-57.06, 38.95) | 2.89 (-74.65, 71.84) | 0.71 (-58.41, 37.8) | 1.96 (-57.04, 39.25) | 1.74 (-72.47, 54.75) | -0.65 (-59.89, 36.67) | AT | 0.48 (-18.79, 17.95) |
| **2.2 (1.32, 3.09)** | 0.22 (-2.1, 2.48) | **2.95 (1.39, 4.49)** | 3.35 (-46.97, 63.9) | 1.7 (-0.09, 3.45) | **3.04 (1.21, 4.85)** | 2.58 (-49.76, 51.13) | 0.43 (-1.87, 2.76) | 1.05 (-36.26, 60.03) | CON |

The upper-right triangle corresponds to MMSE outcomes (row vs. column), while the lower-left triangle corresponds to MoCA outcomes (column vs. row). Statistically significant results are highlighted in bold. MA, manual acupuncture; EA, electroacupuncture; MOX, moxibustion; TN, tuina; MBE, mind-body exercise; TEAS, transcutaneous electrical acupoint stimulation; ACE, acupoint catgut embedding; WA, warm-needling acupuncture; AT, auricular therapy; CON, control group.

**Supplementary Table 12** League table for uMCI subgroup.

| MA | EA | MOX | TN | MBE | TEAS | ACE | WA | AT | CON |
| --- | --- | --- | --- | --- | --- | --- | --- | --- | --- |
| MA | 0.42 (-0.26, 1.11) | 1 (0.31, 1.72) | -0.32 (-1.27, 0.67) | -0.47 (-1.84, 0.86) | — | -0.16 (-1.11, 0.78) | 0.21 (-1.19, 1.59) | 0.47 (-0.44, 1.37) | 1.66 (1.22, 2.11) |
| 1.26 (-0.1, 2.62) | EA | 0.58 (-0.17, 1.35) | -0.73 (-1.73, 0.28) | -0.89 (-2.31, 0.47) | — | -0.58 (-1.62, 0.46) | -0.21 (-1.63, 1.18) | 0.05 (-0.9, 0.99) | 1.24 (0.72, 1.76) |
| 0.7 (-0.47, 1.88) | -0.55 (-1.97, 0.84) | MOX | -1.32 (-2.34, -0.3) | -1.48 (-2.9, -0.1) | — | -1.16 (-2.23, -0.12) | -0.79 (-2.23, 0.61) | -0.53 (-1.51, 0.41) | 0.66 (0.1, 1.19) |
| -0.81 (-2.48, 0.86) | -2.07 (-3.92, -0.24) | -1.51 (-3.21, 0.18) | TN | -0.15 (-1.73, 1.37) | — | 0.16 (-1.11, 1.4) | 0.52 (-1.05, 2.07) | 0.79 (-0.4, 1.94) | 1.98 (1.11, 2.83) |
| 0.01 (-1.12, 1.13) | -1.25 (-2.6, 0.11) | -0.69 (-1.86, 0.48) | 0.82 (-0.86, 2.49) | MBE | — | 0.31 (-1.23, 1.9) | 0.67 (-1.12, 2.54) | 0.94 (-0.54, 2.47) | 2.13 (0.88, 3.44) |
| -1.59 (-4.05, 0.9) | -2.84 (-5.45, -0.26) | -2.3 (-4.74, 0.22) | -0.78 (-3.51, 2.01) | -1.6 (-4.06, 0.9) | TEAS | — | — | — | — |
| -1.05 (-3.48, 1.39) | -2.31 (-5.11, 0.46) | -1.75 (-4.46, 0.94) | -0.25 (-3.2, 2.72) | -1.06 (-3.75, 1.63) | 0.53 (-2.95, 3.97) | ACE | 0.37 (-1.23, 1.96) | 0.63 (-0.58, 1.82) | 1.82 (0.93, 2.73) |
| 0.95 (-1.51, 3.43) | -0.31 (-2.86, 2.3) | 0.25 (-2.2, 2.74) | 1.75 (-0.96, 4.53) | 0.94 (-1.5, 3.44) | 2.53 (-0.73, 5.81) | 2.01 (-1.45, 5.49) | WA | 0.26 (-1.28, 1.79) | 1.45 (0.15, 2.78) |
| 1.05 (-0.86, 2.94) | -0.22 (-2.25, 1.85) | 0.34 (-1.57, 2.26) | 1.85 (-0.41, 4.11) | 1.03 (-0.87, 2.93) | 2.63 (-0.27, 5.51) | 2.1 (-0.99, 5.2) | 0.08 (-2.81, 2.97) | AT | 1.19 (0.41, 1.99) |
| 2.17 (1.38, 2.98) | 0.92 (-0.19, 2.02) | 1.47 (0.62, 2.33) | 2.98 (1.52, 4.46) | 2.16 (1.38, 2.97) | 3.76 (1.41, 6.09) | 3.23 (0.66, 5.8) | 1.23 (-1.12, 3.54) | 1.13 (-0.59, 2.85) | CON |

The upper-right triangle corresponds to MMSE outcomes (row vs. column), while the lower-left triangle corresponds to MoCA outcomes (column vs. row). Statistically significant results are highlighted in bold. MA, manual acupuncture; EA, electroacupuncture; MOX, moxibustion; TN, tuina; MBE, mind-body exercise; TEAS, transcutaneous electrical acupoint stimulation; ACE, acupoint catgut embedding; WA, warm-needling acupuncture; AT, auricular therapy; CON, control group.

**Supplementary Table 13** SUCRA rankings for MMSE scores by intervention population.

| Rank | aMCI | SUCRA (%) | uMCI | SUCRA (%) |
| --- | --- | --- | --- | --- |
| 1 | MOX | 72.64 | MBE | 81.77 |
| 2 | MA | 65.53 | TN | 79.25 |
| 3 | TN | 55.85 | ACE | 72.03 |
| 4 | MBE | 53.41 | MA | 65.22 |
| 5 | ACE | 51.66 | WA | 53.66 |
| 6 | AT | 45.23 | EA | 41.08 |
| 7 | WA | 41.24 | AT | 39.73 |
| 8 | EA | 41.12 | MOX | 16.9 |
| 9 | CON | 23.34 | CON | 00.36 |

MA, manual acupuncture; EA, electroacupuncture; MOX, moxibustion; TN, tuina; MBE, mind-body exercise; TEAS, transcutaneous electrical acupoint stimulation; ACE, acupoint catgut embedding; WA, warm-needling acupuncture; AT, auricular therapy; CON, control group.

**Supplementary Table 14** SUCRA rankings for MoCA scores by intervention population.

| Rank | aMCI | SUCRA (%) | uMCI | SUCRA (%) |
| --- | --- | --- | --- | --- |
| 1 | TEAS | 74.42 | TEAS | 88.72 |
| 2 | MOX | 73.7 | TN | 79.85 |
| 3 | MA | 60.68 | ACE | 79.64 |
| 4 | TN | 57.32 | MA | 60.61 |
| 5 | ACE | 53.4 | MBE | 60.56 |
| 6 | MBE | 51.67 | MOX | 37.96 |
| 7 | AT | 47.3 | WA | 34.62 |
| 8 | WA | 31.16 | AT | 30.89 |
| 9 | EA | 27.99 | EA | 23.75 |
| 10 | CON | 22.36 | CON | 03.4 |

MA, manual acupuncture; EA, electroacupuncture; MOX, moxibustion; TN, tuina; MBE, mind-body exercise; TEAS, transcutaneous electrical acupoint stimulation; ACE, acupoint catgut embedding; WA, warm-needling acupuncture; AT, auricular therapy; CON, control group.
